# Understanding Universal Reorientation Behavior of Active Janus Colloids

**DOI:** 10.1021/acsnano.6c03119

**Published:** 2026-07-27

**Authors:** Kelly Henze, William E. Uspal, Martin Wittmann, Juliane Simmchen

**Affiliations:** † Physical Chemistry, 9169 TU Dresden, 01069 Dresden, Germany; ‡ Department of Mechanical Engineering, 3949 University of Hawai’i at Ma̅noa, Honolulu, Hawaii 96822, United States; § Leibniz-Institut für Polymerforschung Dresden e.V., Dresden 01069, Germany; ∥ International Institute for Sustainability with Knotted Chiral Meta Matter (WPI-SKCM^2^), Hiroshima University, 1-3-1 Kagamiyama, Higashi-Hiroshima, Hiroshima 739-8531, Japan; ⊥ Pure and Applied Chemistry, University of Strathclyde, Glasgow G1 1XL, U.K.

**Keywords:** active colloids, Janus particles, orientation, wall interactions

## Abstract

The dynamics of Janus particles near a wall, including velocity, MSD, propulsion mechanisms, and direction, are an ongoing subject of research. However, the orientation of these particles while swimming near a solid boundary remains largely unexplored. In particular, a systematic analysis of the factors influencing their behavior across a broad range of materials and fuels is missing. Here, we present and explore the intriguing observation that the cap orientation is constant across a wide range of material-fuel combinations. Our findings showed a stable orientation at an angle (θ) of around 90°, indicated by the vector from the catalytic “pole” to the inert pole, with respect to the wall normal. To understand this behavior, we apply a continuum model of neutral self-diffusiophoresis near an osmotic-responsive planar wall. This model predicts sliding states θ ≳ 90° (≲90°) for inert-forward (cap-forward) particles. The experimentally confirmed angles of around 90° correspond to these sliding states and persist even as the active materials of the Janus particles and the fuels, and thereby the respective *b* values in the model, are changed. Moreover, an analytical model reveals a critical role for the hydrodynamic force quadrupole in biasing particle tilt toward the wall. An approximate angle of ≲90° is also observed for “cap-forward” particles under certain conditions on the osmotic responsiveness of the wall.

## Graphical abstract

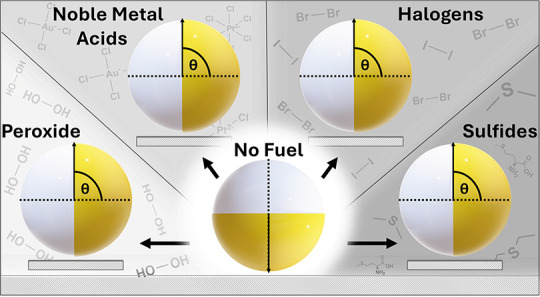

Pioneers in the field of origin-of-life research approach the question of fundamental distinctions between living and nonliving systems generally from a chemical perspective. This emphasizes that understanding life-like behavior entails more than simply examining isolated components.^1–3^ The concept of system chemistry is central to this comprehension; it shifts the focus to how dynamic networks of molecules interact, evolve, and give rise to emergent properties. This system-level perspective may provide a promising bridge between synthetic active matter and the complex functions of living systems. Active matter is an emerging field that investigates the differences between living and nonliving systems from a statistical physics perspective.^4^ Integrating the physical principles of out-of-equilibrium dynamics with chemical reactivity has the potential to increase our understanding of the correlations between complex chemical and fluid dynamic processes. Unlike living systems, which rely on cascades of chemical reactions, most artificial active systems are rather simple so that individual effects can be studied on a physical basis. The most common example is the Pt Janus particle, which propels itself through the catalytic decomposition of H_2_O_2_.^5^ Its motion has been extensively studied in terms of direction, orientation, wall interactions, and collective behavior.^6–10^ Recent developments have broadened the range of chemical fuels beyond H_2_O_2_ degradation, now including diverse classes of reactions such as noble metals acids,^11,12^ halogens,^13,14^ and sulfides. These alternatives promise more diverse and potentially less toxic propulsion mechanisms while maintaining or even increasing efficiency.^15^ Although studies have begun to investigate how different chemical reactions influence propulsion,^11,14,16^ our understanding of how various factors affect the dynamics of active matter remains limited.

Our study investigates the unique swimming behaviors across various driving reactions, with a particular focus on the origin of their consistent swimming angles. By comparing different reaction classes, we analyze how swimming angles vary across different mechanisms, swimming directions, and velocities. In addition to experimental work, we also approach this topic from a theoretical perspective by modeling how the swimming angle depends on the microscopic properties of the particle and the nearby solid substrate.

## Results and Discussion

Understanding the factors that affect the reorientation angle and studying different Janus particle systems and their behaviors are key steps toward forming a hypothesis about what causes the reorientation angle (θ). Here, θ = 0 is defined as the particle orientation where the symmetry axis is perfectly orthogonal to the substrate, as illustrated in Figure 1. Through various modeling approaches and simulations, significant progress has been made toward understanding the variables that affect the behavior of active matter.^6^ One of the most powerful methods for interfacially driven active particles is the boundary element method (BEM).^17^ In the context of self-phoretic particles, BEM resolves the self-generated scalar (e.g., concentration, electrostatic potential) and hydrodynamic fields that govern particle motion. Notably, the particle interacts with its surrounding environment mediated by these fields, such as when a solid surface confines the reaction product. BEM has proven highly effective at predicting swimming properties, including the swimming angle near a wall, for linear models of self-phoresis. By refinement of this method,^6,18^ it was possible to analyze the key factors affecting particle orientation near a substrate. The main influences identified include bottom heaviness, the hydrodynamic stress tensor (i.e., hydrodynamic interactions with the substrate), the density of solute distribution, and the chemiosmotic contributions resulting from interactions with the wall, as well as higher-order effects from the coupling of hydrodynamic and chemical confinements (see Figure 1).

**Figure 1:**
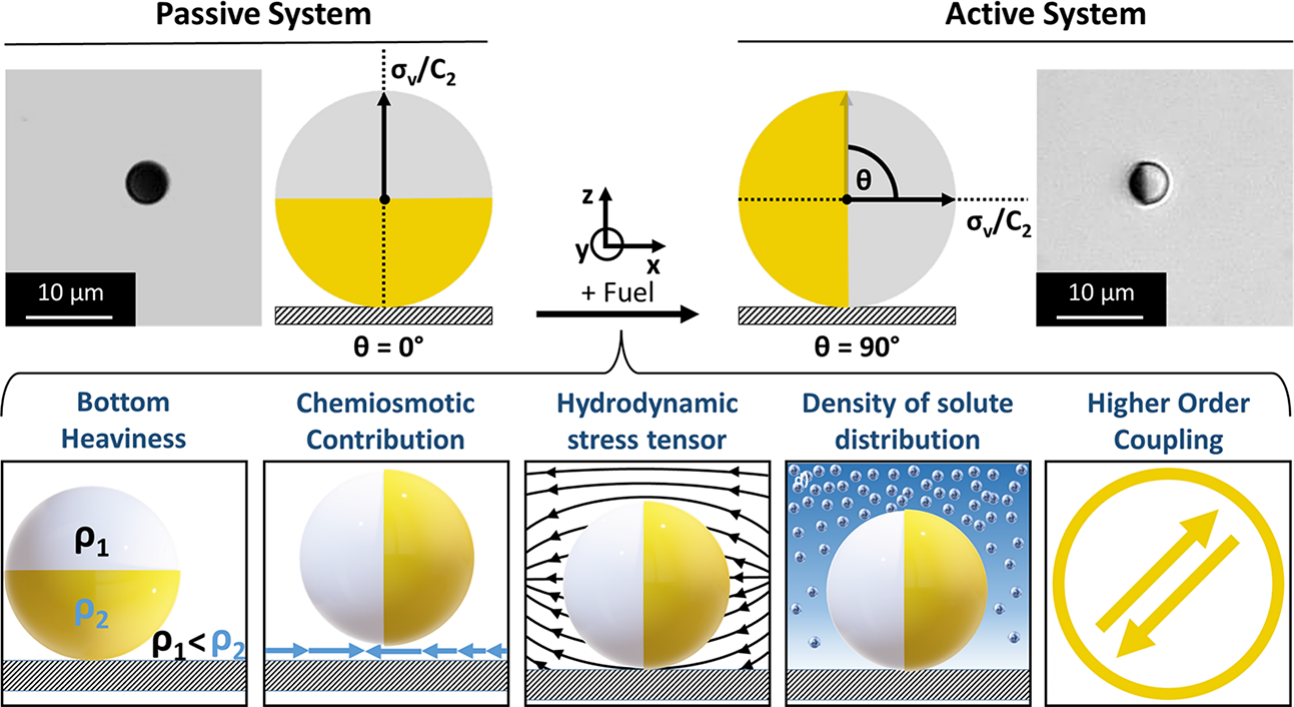
Factors influencing the reorientation process from a passive particle to an active particle near a wall.

To assess how different fuel-driven chemical reactions affect microscale swimming parameters, such as velocity, direction, and changes in particle orientation from passive to active states, we first introduce our experimental system and then provide a theoretical analysis to support our experimental data.

We begin with the well-known system of catalytic (H_2_O_2_) degradation, analyzing the emergence of the well-known equilibrium swimming orientation with various catalytic materials. Next, we broaden our insights to explore different fuel-driven reactions. An overview of the variety of microswimmer systems analyzed in the experimental part section is presented in Table 1.

**Table 1:** Overview of Experimental Analyzed SiO_2_-Based Janus Microswimmer Systems

active metal	fuel	swimming direction	reaction
Pt	H_2_O_2_	inert forward	catalytic^5^
Ag	H_2_O_2_	cap forward	catalytic/redox (oxidation) with ionic products (Ag^+^)^19^
Cu	H_2_O_2_	cap forward	catalytic/redox (oxidation) with ionic products (Cu^2+^)^7^
Ag	HAuCl_4_	cap forward	galvanic exchange reaction
Cu	HAuCl_4_	cap forward	galvanic exchange reaction^16^
Ag	H_2_PtCl_6_	cap forward	galvanic exchange reaction
Cu	H_2_PtCl_6_	cap forward	galvanic exchange reaction^12^
Ag	I_2_	cap forward	redox reaction with solid product (AgI (s))
Cu	I_2_	cap forward	redox reaction with solid product (CuI_2_ (s))^14^
Ag	Br_2_	cap forward	redox reaction with solid product (AgBr (s))
Cu	Br_2_	cap forward	redox reaction with ionic product (CuBr_2_ (aq))^14^
Cu	DMS	cap forward	redox reaction with solid product (Cu_2_O (s))^20^

### Influence of Gravity

Despite H_2_O_2_ being a classic and well-researched fuel in active matter, many of the intricate details on propulsion and motility are not yet understood. The overall reaction

$$2H_{2}O_{2}\to 2H_{2}O+O_{2}$$ 1

indicates that no charges are involved, which, however, does not exclude the possibility of intermediate products of ionic character.^21–23^ The mechanism for peroxide-driven Pt Janus colloids is typically assumed to be self-diffusiophoresis with a certain influence of self-generated ions.^22,23^ The asymmetric solute concentration is generated by the particle itself through the catalytic degradation of H_2_O_2_ on a Pt surface. The created O_2_ generates a tangential concentration gradient in the surrounding H_2_O. Irrespective of the nature of the solutes (neutral or ionic), the interactions generate a fluid flow in a thin layer around the particle surface.^11,14,24^ Here, we use different catalysts of different nominal densities (see Figure 2) to study the influence of gravity on the orientation behavior.

**Figure 2:**
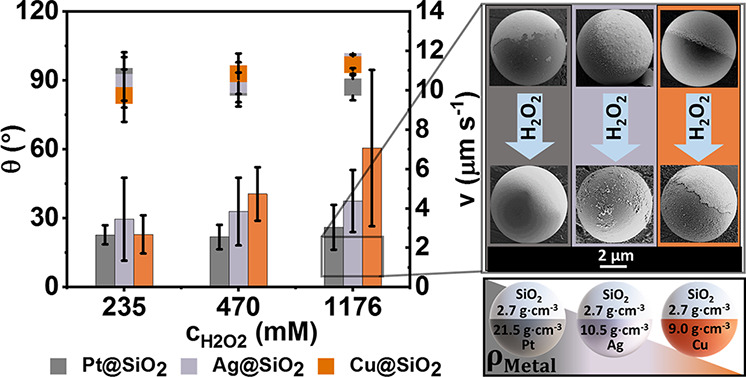
Angle and velocity values for SiO_2_ Janus particles with different metal caps at a concentration range of 235–1176 mM H_2_O_2_, as well as SEM images of the particles before and after their reaction with 1176 mM H_2_O_2_. Additionally, a visualization of the density relationship among the metals is included.

We start by considering Janus particles with a traditional Pt cap in H_2_O_2_. Throughout the catalytic process,^25,26^ the cap remains unchanged (see Figure 2).

The swimming direction is away from the catalytic cap, and we observe increasing velocity with increasing fuel concentration, which is in agreement with previous studies.^7^ Our analysis of swimming behaviors also considers how a particle orients its cap during the propulsion reaction, an aspect that has received limited attention in the literature.^6,27,28^ By depositing a 30 nm Pt layer on one hemisphere of the SiO_2_ particle, we shift the center of gravity. The theoretical mass of an ideal half-cap of Pt is calculated to be 2.55 × 10^–11^ g (see Table S1). Thus, the cap of a bottom-heavy particle turns toward the substrate in the passive state (no fuel addition). As shown in Figure S2, experimentally, we can see a highly stable angle of 0° in the passive state, which confirms earlier findings.^29^ When hydrogen peroxide is added to the Pt@SiO_2_ particles, they become active and reorient so that the symmetry axis of the particle is now parallel to the substrate.^6^ The angle analysis, described in the SI, shows that the active particles exhibit an angle of approximately 90° when propelling. In the long-duration experiments shown in Video S1, the reorientation of the Pt@SiO_2_ particles begins immediately after the fuel is added and remains consistent until the fuel is consumed. This is clearly visible in Video S6, as the particle stops moving once the fuel is consumed, when the particle reorients back to its initial orientation at an angle of approximately 0°. The orientation of an actively swimming Pt@SiO_2_ particle remains constant across a concentration range from 235 to 1176 mM H_2_O_2_ (see Figure S8), despite concentration-dependent increasing velocity. Upon exchanging the Pt catalyst for Ag, the catalytic decomposition of H_2_O_2_ happens on the Ag hemisphere. A key distinction between Ag@SiO_2_ particles and their Pt@SiO_2_ counterparts is that they swim with their metal caps forward. This swimming direction is driven by the additional oxidation of Ag through H_2_O_2_, a potent oxidizing agent,^25,29,30^ which oxidizes Ag to Ag^+^ ions while forming OH^–^ ions and radicals (see eq 2).^57^

$$H_{2}O_{2}+\mathrm{Ag}\to {\mathrm{OH}}^{-•}+{\mathrm{OH}}^{•}+{\mathrm{Ag}}^{+}$$ 2

Notably, the varying diffusion constants of these ions (*D*_Ag^+^_ = 1.648 × 10^–9^ m^2^·s^–1^,^30^ *D*_OH^–^_ = 5.273 × 10^–9^ m^2^·s^–1^ ^30^) create an electric field that induces an electro-osmotic flow from the Ag cap toward the inert silica hemisphere. This additional process, called ionic self-diffusiophoresis, enhances the velocity of the Ag-capped particles in comparison to those capped with Pt, as shown in Figure 2, and plays a crucial role in directing the swimming motion of the particles, ultimately causing them to advance cap first.^31^ Furthermore, an analysis of the swimming behavior and velocity of Cu@SiO_2_ particles reveals similarities to the Ag@SiO_2_ particles. They also exhibit cap-forward swimming behavior and reach a peak velocity of 7.06 μm·s^–1^ at a concentration of 1176 mM H_2_O_2_, surpassing the two particle systems shown before (see Figure 2). This outcome stems from the catalytic decomposition of H_2_O_2_ on the Cu side of the particle and the oxidation of copper by H_2_O_2_. The scanning electron microscopy (SEM) analysis of all particles, before and after the reaction with 1176 mM H_2_O_2_ in Figure 2, along with close-up images of the caps of the particles after the reaction (see Figure S6), reveals a clear transformation. The silver cap has been nearly completely consumed. At the same time, the Cu particles exhibit a much rougher cap than before the reaction, indicating an additional oxidation process of the Cu caused by H_2_O_2_.

Considering the different densities for Ag and Cu are 10.49 and 8.96 g·cm^3^, respectively, both are higher than that of SiO_2_ (2.65 g·cm^3^) but lower than that of Pt (21.45 g·cm^3^). This makes the particles slightly less bottom-heavy in their passive states, but the behavior still resembles that of the Pt Janus particles (see Figure S2). However, similar to the Pt@SiO_2_ particles, the Ag@SiO_2_ and Cu@SiO_2_ particles reorient after activation to approximately 90° and maintain this swimming angle consistently (see Figure S8) over concentrations ranging from 235 to 1176 mM of H_2_O_2_.

In conclusion, different materials used for the caps of the Janus particles (Pt, Ag, and Cu) exhibit slightly different reaction mechanisms, resulting in varying swimming directions. Notably, the Pt cap acts as a perfect catalyst and is not modified during the process, while higher velocities can be achieved with Ag and Cu caps that are partially involved in the reaction and produce (intermediate) metal ions. Despite the differing densities of the catalytic caps, consistent orientation behavior is observed. This excludes gravity as a dominant factor influencing the orientation of the particles.

### Broadening Fuel Variations

To understand the factors influencing the reorientation process of Janus particles, we analyze a broader range of Janus swimmer systems by expanding the fuel options, which leads to different reaction mechanisms. First, we shift to **noble-metal acids** as fuels, which utilize galvanophoretic propulsion via a redox mechanism. Due to their standard potentials, noble-metal acids, such as HAuCl_4_ and H_2_PtCl_6_, can react with less noble-metal caps, such as Cu or Ag. For example, when a solid copper cap is used in HAuCl_4_, it dissolves, producing Cu^2+^ ions. Meanwhile, Au from the AuCl_4_^–^ ions is deposited as a solid onto the Janus particle surface (see Figure 3).^11,12^

**Figure 3:**
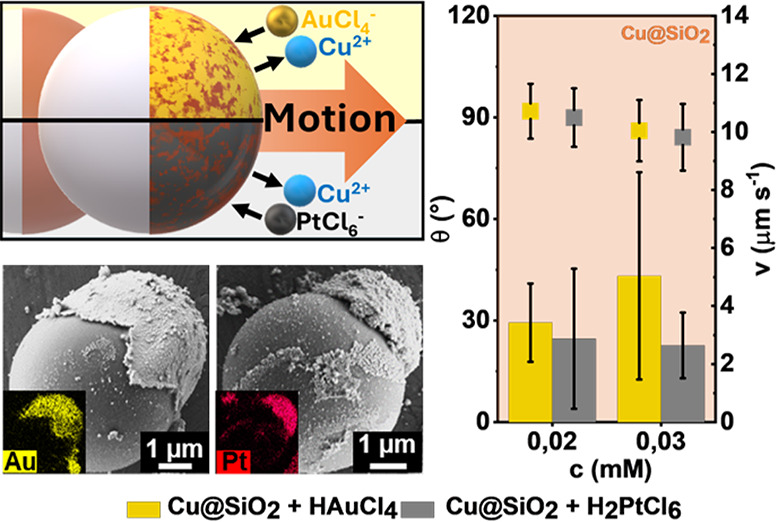
Schematic overview of the reaction between noble-metal acids and a Cu@SiO_2_ particle, including the effects on their morphology and cap composition after reacting with 0.5 mM HAuCl_4_ or H_2_PtCl_6_, respectively. This is followed by experimental angle and velocity results for both noble-metal fuels with Cu@SiO_2_ at concentrations of 0.02 and 0.03 mM.

This process is evidenced by the SEM and energy-dispersive X-ray spectroscopy (EDX) measurements shown in Figure 3 for copper. It is visible that the cap morphology has changed, as well as its composition. The same changes can also be seen for Ag@SiO_2_ particles, which reacted with noble-metal acids, shown in Figure S9. Regardless of the cap metal or the noble-metal acid used, the particles swim with their caps forward. This can be attributed to differing diffusion coefficients of the resulting ions, such as Cu^2+^ and Cl^–^ (*D*_Cu^2+^_ = 0.71 × 10^–9^ m^2^·s^–1^,^30^ *D*_Cl^–^_ = 2.03 × 10^–9^ m^2^·s^–1^ ^30^), which generate an electric field that causes ionic current around the particle. This ionic current, in turn, creates a fluid flow around the particle, propelling it forward. According to Feuerstein et al.,^11^ propulsion arises from a self-electrophoretic mechanism with a self-diffusiophoretic contribution. These swimmers, particularly the reaction with HAuCl_4_, demonstrate significantly higher efficiencies compared to H_2_O_2_. Specifically, for the HAuCl_4_ fuel, Cu@SiO_2_ particles demonstrate a velocity of 5 μm·s^–1^ at a concentration of 0.03 mM (see Figure 3, while Ag@SiO_2_ particles show a velocity of 4.7 μm·s^–1^ at the same concentration (see Figure S9). However, the Ag@SiO_2_ particles show no directed motion when reacting with H_2_PtCl_6_. This is indicated by their low velocities of around 2 μm ·s^–1^, which are similar to the values for Brownian motion and MSD curves in (Figure S10), which resemble Brownian motion and do not differ significantly from those without fuel. This can be explained by the proximity of the reduction potentials of Ag (*E*_black_ = 0.799 V^30^) and PtCl_6_^2–^ (*E*_red_ = 0.74 V^12^). This creates a redox potential near zero, which in turn produces insufficient force to move the particle forward. However, the reorientation angles of the Ag@SiO_2_ particles in H_2_PtCl_6_ remain relatively stable at around 90° (see Figure S11). Following our analysis of noble-metal acid fuels, we examine reorientation angles close to 90°, with high-angle stabilities at different concentrations. We find that Janus particles with Cu and Ag metal caps exhibit similar reorientations in H_2_O_2_ and noble-metal acids, which is notable, considering that they perform completely different reaction mechanisms.

In the following, we broaden the fuel options further by using **halogens** such as I_2_ and Br_2_. The reaction that occurs depends on the combination of the cap material and fuel used. Cu, as a cap material, reacts with the halogens to form either CuI or CuBr (see Figure 4), as reported in our previous work.^14^

**Figure 4:**
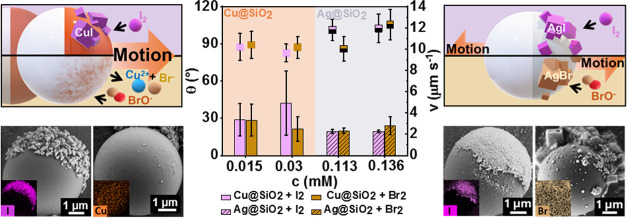
Schematic overview of the reaction between halogens and Cu@SiO_2_ as well as Ag@SiO_2_ particles, including the effects on their morphology and cap composition after reacting with 0.5 mM I_2_ or Br_2_, respectively. Followed by experimental angle and velocity results for both halogen fuels at concentrations of 0.015 and 0.03 mM for Cu@SiO_2_ and 0.113 and 0.136 mM for Ag@SiO_2_.

Using a silver cap instead of copper in this fuel system results in the formation of insoluble products AgI and AgBr. This is demonstrated in the SEM and EDX analysis as shown in Figure 4. Furthermore, the XRD analysis in Figure S12 supports this observation, as the measured diffractograms of the sample closely match the theoretical pattern for AgI and AgBr. However, the products and ions generated around the particles differ strongly for the various reactions. Comparing Cu@SiO_2_ and Ag@SiO_2_ particles in halogen fuel shows that Cu@SiO_2_ exhibits slightly higher velocities with both halogen fuels; the absence of increasing velocity with higher bromine concentration is consistent with our earlier publication.^14^ In contrast, Ag@SiO_2_ particles require approximately four times higher concentrations of fuel to initiate propulsion (see Figure 4), and their velocity remains low. However, the calculated MSD curves (see Figure S13) clearly show ballistic motion in response to the reaction with halogens. Notably, particles propelled by I_2_ tend to swim with their inert hemisphere moving forward. In contrast, particles reacting with Br_2_ generally swim with their Ag cap leading. Moreover, the velocity of the particles reacting with Br_2_ increases from 2.3 to 2.8 μm·s^–1^ as the concentration rises, as displayed in Figure 4. This can be explained by the formation of the insoluble product AgBr, which affects the overall salt concentration. For a more detailed discussion of the copper system, please refer to ref 14. Despite all differences in reactions and propulsion systems, the swimming angles in the active state remain constant around 90° for the four different reactions (see Figure 4). The high-angle stability in Figure S14 thus confirms behavior comparable to that of H_2_O_2_ and noble-metal acid fuels.

Apart from the above-presented redox fuel systems, Cu can participate in a broader range of reactions with various other fuel types. One notable example is its interaction with **organosulfur compounds**.^32,33^ We found that Cu@SiO_2_ particles have demonstrated the ability to propel in aqueous disulfides, as illustrated in Figure 5.^20^

**Figure 5:**
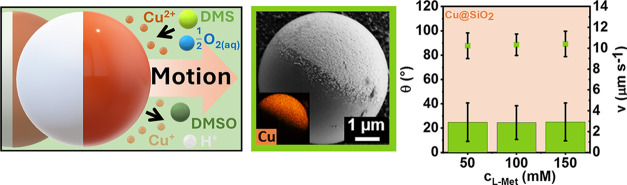
Schematic overview of the reaction between DMS and Cu@SiO_2_, including the effects on their morphology and cap composition after reacting with 500 mM DMS, respectively, followed by experimental angle and velocity results for concentrations of 50, 100, and 150 mM.

The reactions involving these sulfur-containing compounds have yet a different mechanism, detailed in ref 20. The overall reaction evolves around the oxidation of DMS to DMSO with a catalytic influence of Cu ions. The results from SEM measurements suggest that the Cu cap partially dissolves, rather than completely dissolving, indicating that Cu ions may enter solution and form intermediate complexes with the sulfur compounds. Analysis of the MSD curves indicates that the particles exhibit active motion with the organosulfur fuel. As the concentration of the fuel increases, the velocity of the particles also increases,^20^ but the measurements presented here are at 50 and 1000 mM and 150 mM, where the plateau is reached, and we observe only a minimal increase. The reorientation angle (θ) shows values between 82 and 92°, which align with previous results for other microswimmer systems despite the very different chemical propulsion mechanism. Additionally, when examining the angle stability of the Cu@SiO_2_ particles in organosulfur fuels, it is evident that they maintain it particularly around the value of 90° (see Figure S16). This shows that multispecies reactions can also lead to consistent swimming orientations.

### Theoretical Contribution

To better understand the ubiquity of the θ ≈ 90° swimming angle, we deploy a widely used continuum model for neutral self-diffusiophoresis.^34^ In this model, the cap of the particle catalyzes a reaction involving a “fuel” molecule that is plentiful in the suspending medium to produce an uncharged reaction product (“solute”). The Péclet number Pe ≡ *U*_0_ *R*/*D*, where *R* is the radius of the particle, *U*_0_ is a characteristic velocity of the particle-sourced fluid flow, and *D* is the diffusion coefficient of the product molecule, is taken to be very small. Therefore, the concentration (number density) of the reaction product *c*(**x**) is quasi-steady and governed by Laplace’s equation, ∇^2^ *c* = 0. The catalytic cap is assumed to generate product molecules at a constant and uniform rate κ per unit area, such that −*D*[**n** **^**·∇*c*] = κ on the cap, where the normal vector **n** **^** is defined to point into the fluid. The particle moves near a solid planar wall, and *c*(**x**) obeys the boundary condition **n** **^**·∇*c* = 0 on the inert “face” of the particle and on the wall. Additionally, *c*(**x**) vanishes far from the particle. The self-generated surface gradient of the reaction product ∇_||_ *c*, in conjunction with some molecular-scale interaction between the solute and the particle surface, drives an interfacial flow (“surface slip”) **v**_s_ = −*b*∇_||_ *c*, powering particle motion. The solute/surface interaction is encoded as the so-called phoretic mobility *b*, a material-dependent parameter.^35^ Moreover, when the particle moves near a solid substrate, the particle can locally evoke a “chemiosmotic” flow by the same molecular mechanisms.^6,36^ (Here, “osmotic” distinguishes a fixed surface from a freely suspended particle.) The chemi-osmotic interfacial flow drives flow in the bulk solution, coupling back to the particle and affecting its motion. The Reynolds number *Re* ≡ ρ*U*_0_ *R*/μ, where ρ is the fluid density and μ is the dynamic viscosity, is assumed to be very small. Therefore, the fluid velocity **u**(**x**) is governed by the Stokes equation −∇*p* + μ∇^2^ **u** = 0, where *p*(**x**) is the pressure. The fluid velocity obeys the boundary conditions **u**(**x**_s_) = **v**_s_(**x**_s_) on the planar wall and **u**(**x**_s_) = **U** + **Ω** × (**x**_s_ – **x**_p_) + **v**_s_(**x**_s_) on the particle surface, where **x**_s_ is a position at a fluid/solid interface, and **U** and **Ω** are the unknown translational and angular velocities of the particle. Additionally, the particle is subject to a short-ranged repulsive force from the wall and, where indicated, a gravitational force. The contributions of these forces to the particle velocity can be calculated within the standard hydrodynamic resistance framework, as discussed in Materials and Methods section. Since the particle motion is overdamped in the regime Re ≪ 1, the sum of forces and the sum of torques on the particle both vanish. These two conditions close the system of equations for **U** and **Ω**. The complete specification of the model is given in Materials and Methods section.

The parameter *b*, which is expected to be different between the two “faces” of the particle and between the particle and substrate, can be considered an effective parameter that depends on the nature of the solute/surface interaction, conditions in the solvent (e.g., salt concentration, pH), surface functionalization, etc. Accordingly, a systematic examination of sliding angle as a function of the surface mobilities of the two particle “faces” and of the solid substrate would yield insight into the apparent ubiquity of θ ≈ 90°. Does this value dominate most of the parameter space? Or is it restricted to a region of that space, constraining experimentally relevant surface mobility parameters?

Here, we aim to address these questions. First, we introduce the governing parameters and physical assumptions; the details of our model are in Materials and Methods section. The inert face of the particle has phoretic mobility *b*_i_ *b*_0_, the catalytic face has phoretic mobility *b*_c_ *b*_0_, and the wall has phoretic mobility *b*_w_ *b*_0_. Here, *b*_0_ > 0 is a characteristic phoretic mobility with units of m^5^ s^–1^, while *b*_i_, *b*_c_, and *b*_w_ are dimensionless. The signs of *b*_i_, *b*_c_, and *b*_w_ encode whether the effective interaction is attractive (positive) or repulsive (negative). Without loss of generality, one of the parameters *b*_i_, *b*_c_, and *b*_w_ can be taken to have unit magnitude. Additionally, we consider two limiting scenarios: (i) the particle is neutrally buoyant (gravitational force negligible) and can move in three dimensions and (ii) the particle is effectively confined to near-surface motion by gravity (heaviness dominant over activity). Scenario (ii) is most commonly realized in experiments, including all experiments in this article, but scenario (i) can be studied with particles with low-density cores.^27,37,38^ In all cases, we assume bottom heaviness is negligible, and therefore, our results can be interpreted as applying to particles with thin hemispherical caps.

Given the above assumptions, the dominant parameters for determination of the sliding angle are two ratios that involve all three of *b*_i_, *b*_c_, and *b*_w_, e.g., *b*_i_/*b*_c_ and *b*_w_/*b*_c_ (see discussion in Materials and Methods). We further assume that the surface mobility of the wall, *b*_w_, never has a larger magnitude than both *b*_i_ and *b*_c_. (A very strong surface mobility on the wall generally leads to “cap-up” or “cap-down” motionless hovering states.) Notably, our results include and extend the results from ref 18, which assume a neutrally buoyant particle near a no-slip wall (*b*_w_ = 0).

#### Repulsive Average Surface Mobility (Inert-Forward)

We first consider scenarios in which the particle has repulsive (negative) average surface mobility, i.e., *b*_c_ + *b*_i_ < 0. These particles will exhibit “inert-forward” motion in bulk liquid (free space),^39^ and are expected to have the same character of motion when moving near solid surfaces, except under unusual circumstances (e.g., strong attractive chemiosmotic response). In this work, the only inert-forward system under experimental consideration is the widely used Pt@SiO_2_ in H_2_O_2_ (see Table 1).

In Figure 6a,b, we consider a neutrally buoyant particle and show the presence (colored circle) or absence (cross) of a stable steady state (fixed point attractor) with *h*/*R* < 6 as a function of *b*_i_ and *b*_w_, taking *b*_c_ = −1. The color indicates the numerical value of the steady angle in degrees. In these plots, the phoretic mobility has the highest magnitude on the cap, since *b*_i_ and *b*_w_ are varied between −1 and 1. In other words, these particles are “cap-dominant” concerning the phoretic mobility. Since the trailing cap will have a larger magnitude slip than the leading inert face, they are expected to be hydrodynamic “pushers.” As will be discussed, previous literature suggests that Pt@SiO_2_ in H_2_O_2_ swimmers belong in the cap-dominant class.^6^ It is clear that for a large region of this parameter space, the particle is in a cap-up “hovering” state with θ = 180°, which is a state of no translational motion. However, there is also a large “sliding” region of parameter space (blue-green colors, middle of the plot) in which the sliding angle is between θ ≈ 85° and θ ≈ 125°. (A version of Figure 6a annotated with numerical values is also provided in Figure S17). For all values of *b*_i_ and *b*_w_ shown, the sliding state exhibits motion away from the catalytic cap. There is a sharp, but apparently continuous, transition between “sliding” and “hovering,” and a clearly discontinuous transition between θ ≈ 90° and the absence of a stable steady state. (In this scenario, the particle scatters off the surface.) The inset shows the distribution of sliding angles in the range 5°≤ θ ≤ 175° (to remove hovering states at θ = 0° and θ = 180° from the distribution). The distribution is clearly right-skewed, with a mean angle of 104.6°.

**Figure 6:**
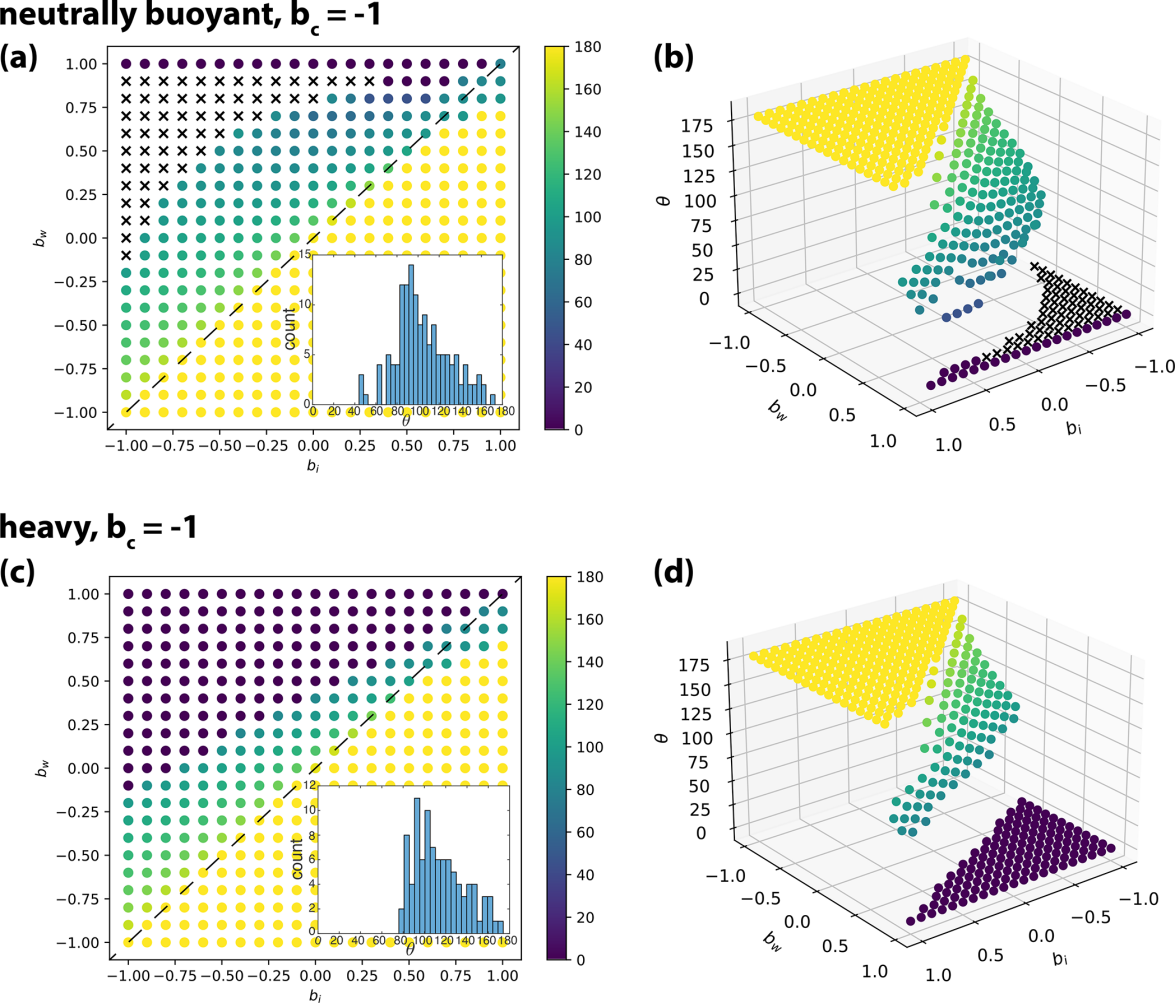
(a) and (b) Sliding angles for a neutrally buoyant particle with *b*_c_ = −1 as a function of *b*_i_ and *b*_w_. (c) and (d) Sliding angles for a gravitationally confined (“heavy”) particle with *b*_c_ = −1 as a function of *b*_i_ and *b*_w_. Insets show the distribution of sliding angles for angles in the range 5° ≤ θ < 175°.

In Figure 6c,d, we show the effect of gravitational confinement. Specifically, the particle is exposed to a gravitational force that contributes a sedimentation velocity to the total velocity of the particle. Since the sedimentation velocity is much larger than the particle’s swimming velocity, the particle is confined to the vicinity of the wall. For all parameters considered, the particles reached a steady state with 1 < *h*/*R* < 1.25; the slight variation is due to the vertical component of the swimming velocity, which depends on particle orientation. We find that the results are qualitatively similar to those shown for the neutrally buoyant particle, except that the black crosses are replaced by blue circles, indicating cap-down, motionless hovering states, and the sliding region is slightly reduced. The mean of the sliding angle is θ = 114.5°.

The plots in Figure 6 are consistent with the apparent ubiquity of θ ≈ 90° for catalytic swimmers in an inert-forward “sliding” state, allowing for some variation of the sliding angle around θ = 90°. Considering the sliding angle function θ­(*b*_i_, *b*_w_) as a surface embedded in a three-dimensional space, the region of the surface with 75° < θ < 125° has gentle curvature. For θ > 125°, moving toward the yellow region of motionless “hovering,” the sliding angle steeply varies with *b*_w_ and *b*_i_. Thus, sliding angles between 125 and 180° are obtained for only small area in the space of (*b*_w_, *b*_i_). The situation for θ < 75° is even more drastic: there are basically no sliding states for these values, except toward the corner of Figure 6a,b (neutrally buoyant particle) with *b*_w_ ≈ 1.0 and *b*_i_ ≈ 1.0.

To obtain more insight into the numerical results, we consider the various contributions to particle rotation shown schematically in Figure 1. The phoretic contribution is due to confinement of the particle-sourced concentration field by the wall. In Figure 7, top left panel, we show the contribution of phoretic rotation to particle rotation θ̇_pr_, obtained as described in Materials and Methods, as a function of angle θ when *h*/*R* = 1.1 and (*b*_i_, *b*_c_) = (1, 0) (black), *h*/*R* = 1.1 and (*b*_i_, *b*_c_) = (0, 1) (red), *h*/*R* = 5.0 and (*b*_i_, *b*_c_) = (1, 0) (green), and *h*/*R* = 5.0 and (*b*_i_, *b*_c_) = (0, 1) (blue). For clarity, each curve is scaled by the maximum absolute value obtained over the interval [0°, 180°]. It is clear that we can approximate θ̇_pr_ as θ̇_pr_ ≈ *f*_pr_(*h*/*R*) (*b*_i_ – *b*_c_) sinθ, with some deviation from this form when the particle is near the wall. Likewise, in the middle panel on the left, one sees that the contribution of wall slip to rotation θ̇_ws_, computed for *b*_w_ = 1.0, is sinusoidal when the particle is far from the wall (green curve, *h*/*R* = 5.0) and approximately sinusoidal when the particle is close to the wall (black curve, *h*/*R* = 1.1.) Therefore, we can write θ̇_ws_ ≈ *f*_ws_(*h*/*R*) *b*_ws_ sin θ. Now, we consider the effect of confinement on the hydrodynamic flow field by the wall (“hydrodynamic interactions”). In the bottom panel on the left, we show the contribution from hydrodynamic interactions to rotation θ̇_hi_, computed as described in Methods, and with the same color scheme as for θ̇_pr_. When the particle is far from the wall (green and blue curves), the form resembles sin2θ, although note that the values for the maximum and minimum have unequal magnitude. Close to the wall (black and red curves), θ̇_hi_ has a complicated dependence on angle. We note that our decomposition of the hydrodynamic and phoretic contributions neglects a higher-order coupling between hydrodynamic and chemical confinements, but this contribution to rotation is small, as discussed in detail in Materials and Methods section.

**Figure 7:**
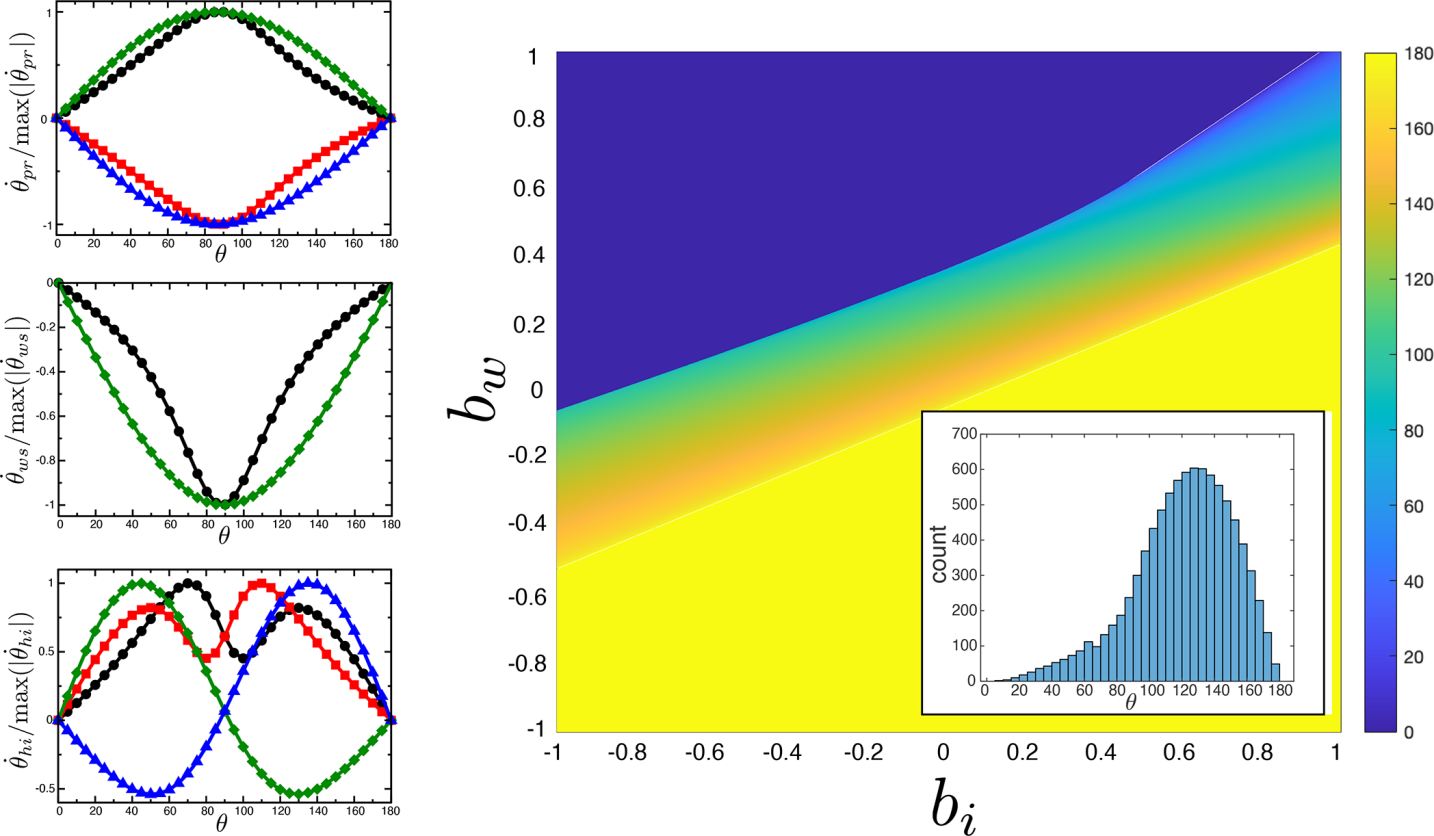
(Top left panel) Contribution of phoretic rotation to particle rotation θ̇_pr_ as a function of angle θ when *h*/*R* = 1.1 and (*b*_i_, *b*_c_) = (1, 0) (black), *h*/*R* = 1.1 and (*b*_i_, *b*_c_) = (0, 1) (red), *h*/*R* = 5.0 and (*b*_i_, *b*_c_) = (1, 0) (green), and *h*/*R* = 5.0 and (*b*_i_, *b*_c_) = (0, 1) (blue). (Middle left panel) Contribution of wall slip to rotation θ̇_ws_ for *b*_w_ when *h*/*R* = 1.1 (black curve) and *h*/*R* = 5.0 (green curve). (Bottom left panel) Contribution from hydrodynamic interactions to rotation θ̇_hi_, with the same color scheme as in the top left panel. (Right) Stable sliding angles obtained with the analytical model as a function of *b*_i_ and *b*_w_ for *b*_c_ = −1. The inset shows the distribution of sliding angles that have 5° < θ < 175°.

Superposing contributions of phoretic rotation, hydrodynamic interactions, and wall slip, we write θ̇ ≈ θ̇_pr_ + θ̇_ws_ + θ̇_hi_. Focusing on the gravitationally confined case, we assume *h*/*R* = 1.1 and estimate *f*_pr_(*h*/*R*) and *f*_ws_(*h*/*R*) from numerical solution to the continuum model (see SI). As discussed in Spagnolie and Lauga,^40^ the contribution from hydrodynamic interactions can be modeled as θ̇_hi_ ≈ θ̇_α_ + θ̇_β_ + θ̇_γ_, where the terms on the right-hand side represent contributions from the hydrodynamic force dipole, source dipole, and force quadrupole, respectively, and terms that are higher order than $O\left({\left(R/h\right)}^{4}\right)$ are neglected. Here, ${\dot{\theta }}_{\alpha }=\frac{3}{16}\alpha_{\mathrm{HI}}(b_{i}-b_{c}){\left(\frac{R}{h}\right)}^{3}\sin\;2\theta$ , ${\dot{\theta }}_{\beta }=-\frac{3}{8}{\left(\frac{R}{h}\right)}^{4}\beta_{\mathrm{HI}}(b_{i}+b_{c})\sin\;\theta$ , and ${\dot{\theta }}_{\gamma }=\frac{3}{8}\gamma_{\mathrm{HI}}(b_{i}+b_{c}){\left(\frac{R}{h}\right)}^{4}\sin\;\theta (1-3\cos^{2}(\theta ))$ . The constants α_HI_, β_HI_, and γ_HI_ are determined from numerical solution of the continuum model, as detailed in the SI, and the sign computed for α_HI_ confirms that the particle has a “pusher” character. Combining the various contributions to θ̇, we obtain θ̇ = sinθ [*A*cos^2^ θ + *B*cos θ + *C*]. The hydrodynamic force quadrupole determines *A*, the force dipole determines *B*, and the quantity *C* has contributions from the source dipole, the force quadrupole, wall slip, and phoretic rotation. To find tilted sliding states θ*, we solve θ̇(θ*) = 0 and evaluate the stability according to the criterion $\frac{d\dot{\theta }}{d\theta }{|}_{\theta *}<0$ . Figure 7, right panel, shows stable states obtained in the model. The model reproduces the band-like structure of the sliding states, the “cliff”-like transition from sliding states with θ ≈ 90° to an absence of tilted slide states, and the continuous transition from tilted sliding states to cap-up (θ = 180°) hovering states. The inset shows the distribution of sliding angles for states with 5° < θ < 175°, which is clearly right-skewed and has a mean angle of 120.8°. The results from the analytical model also have some points of difference with the results in Figure 6. There is a tail of sliding states with θ < 80° that extends to θ = 0°, which occur in a region near *b*_w_ = 1, *b*_i_ = 1. Additionally, the borders of the “band” differ between Figures 6 and 7. However, the semiquantitative agreement we obtain is good for a model based on approximate, far-field analytical expressions, which assumed a fixed particle height. Figure 6 was entirely determined with the boundary element method, which includes near-field interactions, and the BEM calculations allowed for variation of the particle height. The agreement is fortuitous because there was no reason to expect it a priori.

Furthermore, the model provides mechanistic insight into the skewed angle distributions. In particular, we identify a critical role for the force quadrupole term. Figure S21 shows results for the same model with the force quadrupole term set to zero. The distribution of sliding angles is approximately symmetric with respect to 90°, i.e., there is very little bias toward sliding states with θ ≥ 90°. Mathematically, the force quadrupole introduces the quadratic term in the equation for cos θ*. To see the consequences of this, suppose γ_HI_ = 0 (and therefore *C* = 0). The solution for cosθ* would then be cos θ* = −*A*/2*B*, and the equation *A*(*b*_w_, *b*_i_, *b*_c_) = 0, which is linear in the arguments, would determine a line θ* = 90° in the phase diagram. Since arccos satisfies the identity π/2 – arccos­(*x*) = arccos­(−*x*) – π/2, the existence of a sliding state with θ* > 90° (obtained, for example, by starting on the line θ* = 90° and decreasing *b*_w_ by Δ*b*_w_) would imply the existence of a mirror image sliding state with θ* < 90° (obtained, in this example, by starting on the line θ* = 90° and increasing *b*_w_ by Δ*b*_w_).

Since the plots in Figure 6 are “cap-dominant” in terms of phoretic mobility, we also consider the “inert-dominant” case in which *b*_i_ = −1, −1 < *b*_c_ < 1, and −1 < *b*_w_ < 1. These particles are expected to be hydrodynamic pullers, since the phoretic mobility is strongest on the leading face. For the neutrally buoyant case, sliding is only obtained in a small region of parameter space (see Figure S18). However, the situation is different for gravitationally confined particles (see Figure 8). Here, there is a large region of sliding states for repulsive walls (*b*_w_ < 0). For weakly repulsive walls (−0.6 < *b*_w_ < 0), we obtain sliding states with 90° < θ < 125°. For strongly repulsive walls, we obtain sliding states with strong tilt of the cap either away from (θ > 125°) or toward (θ < 50°) the wall. Although we expect inert-forward behavior, the sliding states that have the cap tilted toward the wall have a reversed (cap-forward) motion, as indicated by the diamond symbols. The histogram shows the distribution of sliding angles for angles in the range 5° < θ < 175°. The mean sliding angle for the right half of the distribution (θ > 90°) is 130°, which is somewhat above the mean obtained for a heavy particle with *b*_c_ = −1. Overall, the results for an inert-forward, inert-dominant particle are consistent with experimental observations of the sliding angle if we assume a weakly repulsive chemi-osmotic response of the confining wall. For all parameters considered, the particles had a steady state with 1 < *h*/*R* < 1.25.

**Figure 8:**
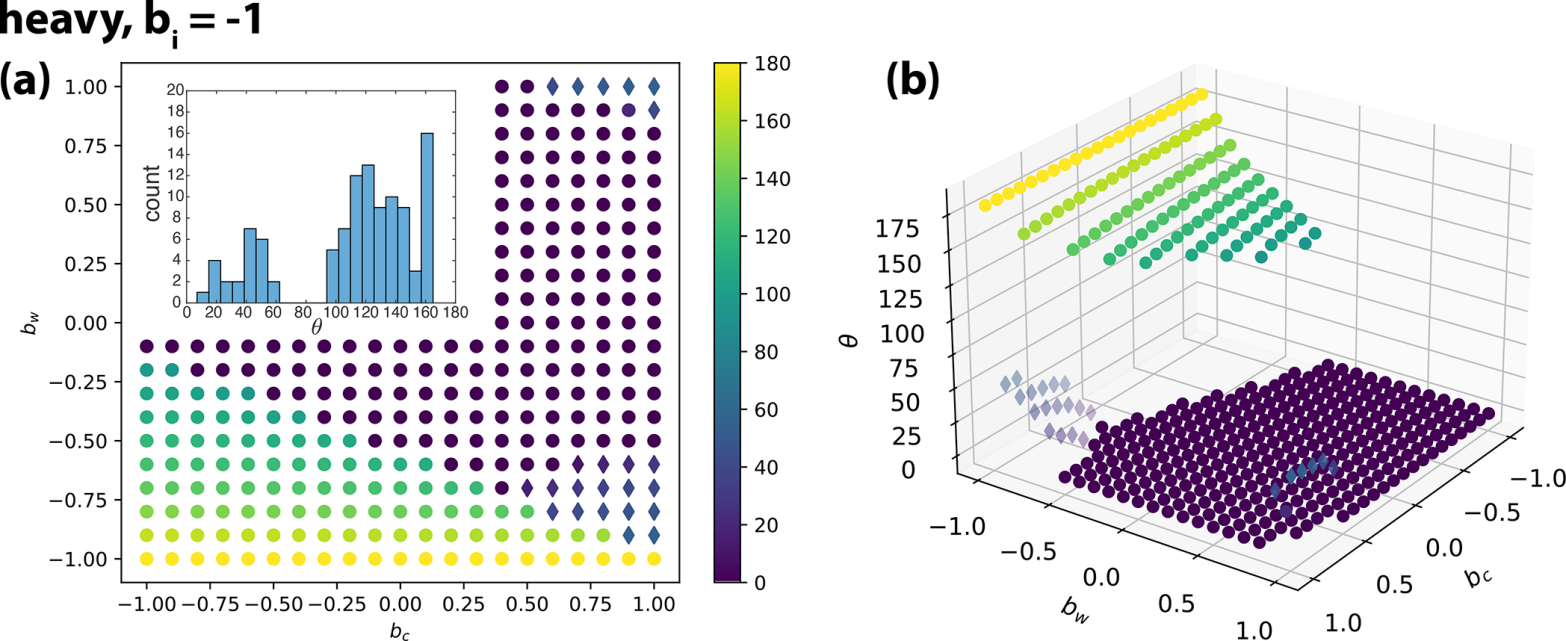
(a) and (b) Sliding angles for a gravitationally confined (“heavy”) particle with *b*_i_ = −1 as a function of *b*_c_ and *b*_w_. Diamond symbols indicate motion-reversed (cap-forward) particles. The inset shows the distribution of sliding angles for angles in the range of 5° ≤ θ < 175°. Note that particles in the left subpopulation θ < 80° have reversed motion.

We note that our previous modeling work on Pt@SiO_2_ Janus spheres has assumed inert-forward, cap-dominant parameters. For instance, in ref 6, the “best-fit” parameters are *b*_c_ = −1, *b*_i_ = −0.3, and *b*_w_ = 0.2. In experiments involving tracer particles in ref 41, *b*_c_ = −1, −0.3 ≤ *b*_i_ ≤ −0.2, and *b*_w_ = *b*_i_ were identified as “best-fit” parameters. The results in Figure 6 indicate that, assuming cap-dominant propulsion, the θ ≈ 90° swimming mode is achieved without fine-tuning the phoretic mobility parameters. Sliding states with θ ≈ 90° are obtained for a wide range of *b*_i_ and *b*_w_, both positive (attractive) and negative (repulsive). Recalling that the cap-dominant particle is a hydrodynamic pusher, our inference of a cap-dominant character for Pt@SiO_2_ is consistent with the inference of a pusher-like hydrodynamic character in other studies.^42,43^

Our findings also have implications for the modeling assumption that *b*_w_ = *b*_i_. Previously, we have made this assumption on the grounds that, for Pt@SiO_2_, the particle core and the substrate are the same material (i.e., glass) and therefore should have the same surface mobility.^41^ However, in Figure 6, this assumption (dashed line) is either on, or close to, the boundary between sliding and cap-up hovering. Thus, our findings suggest that $b_{w}\underset{\approx }{>}b_{i}$ is a better modeling assumption for cap-dominant swimmers. Physically, this assumption may be justified by treatments applied to the substrate to avoid sticking, reducing the effective interaction of the solute and substrate. In Figure 8, where *b*_i_ = −1, the assumption that *b*_w_ = *b*_i_ is clearly in a strongly cap-up region. Therefore, the physically reasonable idea that *b*_w_ ≈ *b*_i_, in conjunction with Figure 6 (cap-dominant) and Figure 8 (inert-dominant), provides additional support for the idea that Pt@SiO_2_ propulsion is cap-dominant.

#### Attractive Average Surface Mobility (Cap-Forward)

Now, we turn to the case when *b*_i_ + *b*_c_ > 0, i.e., the particle has a net average attractive interaction with the product. These particles will move “cap-forward” in the bulk and are expected to generally move cap-forward near solid surfaces. Here, we focus on the case of gravitationally confined particles. (Data for neutrally buoyant particles are presented in the SI). For all parameters considered, the particles had a steady state with 1 < *h*/*R* < 1.07. Overall, we will show that, within the framework of our model, θ ≈ 90° swimming for these particles can be obtained, but under somewhat restrictive (i.e., fine-tuned) conditions. We then discuss implications for the modeling of cap-forward particles.

With the exception of Pt@SiO_2_ in H_2_O_2_, all of the experimental systems considered in this manuscript are cap-forward (see Table 1). However, the cap-dominant or inert-dominant character has received scant attention for these and other active colloidal systems. One way to discriminate between these two scenarios is to determine whether the particle is a hydrodynamic “puller” (i.e., actuated from the leading side) or “pusher” (actuated from the trailing side). Accordingly, cap-forward pullers are cap-dominant, and cap-forward pushers are inert-dominant. We note that Cu@SiO_2_ particles were identified as pullers in a recent study based on a self-electrophoretic model and their rheotactic response.^44^ Alternatively, the cap/inert-dominant character could potentially be inferred from the orientational response of a Janus particle in an externally maintained gradient of either the “fuel” or the product molecule. The face with the higher *b* value would rotate toward the higher concentration. If the average surface mobility is attractive (*b*_i_ + *b*_c_ > 0), this would be the dominant face. Figure 4 in reference 39 provides a helpful schematic relating *b*_i_ and *b*_c_ to the orientational response and the cap-/inert-forward character. It should be noted that this line of reasoning is developed for a particle in free space and omits consideration of a bounding solid substrate (including possible osmotic flows).

We first address the cap-dominant case *b*_c_ = 1, with −1 < *b*_i_ < 1 and −1 < *b*_w_ < 1. The expectation of cap-forward motion is fulfilled for all sliding states we obtain. Considered a surface embedded in a 3D space (see Figure 9a,b), the sliding angle exhibits a complicated dependence on (*b*_i_, *b*_w_), continuously rising from a flat “basin” of cap-down hovering states with θ = 0°. Sliding states with 60° < θ ≤ 80° are observed in a band spanning all values of *b*_i_, with −1 < *b*_w_ < 0.25, adjacent to a jump to cap-up hovering behavior. The mean of sliding angles for angles in the range 5° < θ < 175° (see histogram) is 46°, reflecting the continuous variation of sliding angles from near 0 to 80°. Therefore, θ ≈ 90° sliding is obtained, but with some fine-tuning, not as a dominant feature.

**Figure 9:**
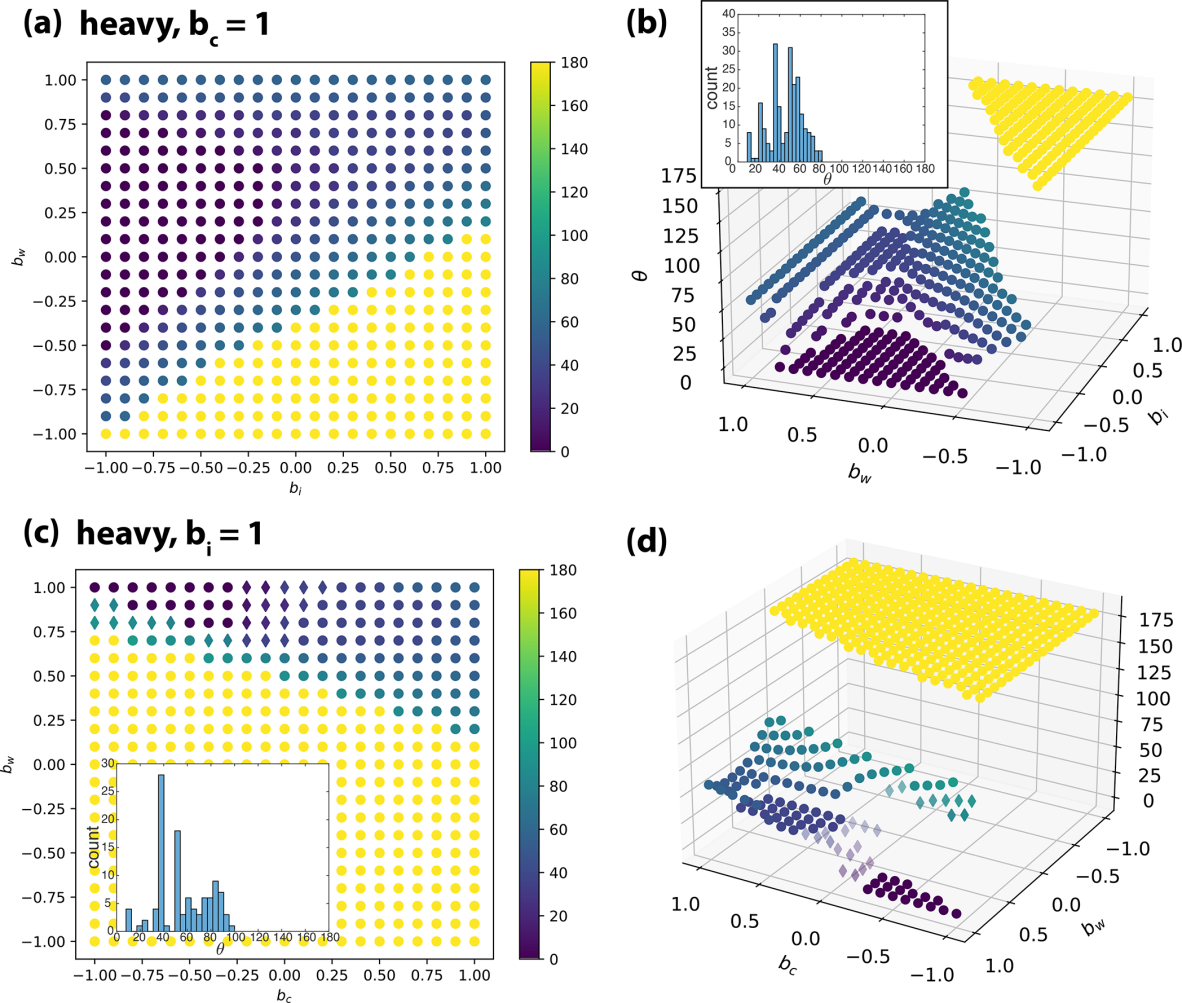
Sliding angles for a gravitationally confined (“heavy”) particle with positive surface-averaged phoretic mobility (*b*_i_ + *b*_c_ > 0). (a) and (b) Sliding angles for *b*_c_ = 1 as a function of *b*_i_ and *b*_w_. (c) and (d) Sliding angles for *b*_i_ = 1 as a function of *b*_c_ and *b*_w_. Diamond symbols indicate motion-reversed (inert-forward) particles. Insets show the distribution of sliding angles for angles in the range 5° ≤ θ < 175°.

Now, we consider inert-dominant particles with *b*_i_ = 1, −1 < *b*_c_ < 1, and −1 < *b*_w_ < 1 (see Figure 9c,d). Although we expect cap-forward behavior, we find that a strong attractive interaction with the wall (large and positive *b*_w_) can lead to motion reversal (inert-forward motion), as indicated by the diamond symbols. Some of these motion-reversed states (*b*_c_ < −0.25) have θ ≈ 90°, although a small region of motion-reversed states (*b*_c_ ≈ 0) has a strong tilt of the cap toward the wall. Turning to the cap-forward states (circles), there is again a band of sliding states with 75° < θ < 100° that spans all values of *b*_c_, here obtained for weak-to-intermediate attractive interactions with the wall (0.25 < *b*_w_ < 0.75). However, there are also sliding states with θ ≈ 40°, obtained for strong attractive interactions with the wall (*b*_w_ ≈ 1). Overall, the mean sliding angle (for sliding angles in the range 5° < θ < 175°) is 56.1°. (If we remove the motion-reversed states from the distribution, the mean angle increases to 59.8°.) Thus, for Figure 9c, we obtain a similar conclusion as for Figure 9a,b: the data are consistent with cap-forward swimming with θ ≈ 90° with some fine-tuning. Here, we need the somewhat restrictive condition of weak-to-intermediate attractive interactions of the product with the wall.

Overall, to obtain cap-forward swimming with θ ≈ 90° in our model, more fine-tuning is required than for inert-forward swimming. The cap-forward swimmers studied here move toward the cap because of charge influences, as indicated by experimental evidence.^45^ Since we use a continuum model of neutral self-diffusiophoresis, this difference in propulsion mechanism may explain the need for additional tuning. It is possible that adjusting the model to more realistically account for charge effects would remove the need for fine-tuning.

## Conclusions

In this study, we produce 5 μm spherical Janus particles made of SiO_2_ and capped with three different metals (Pt,Ag,Cu). We analyze their swimming behavior in different fuels (H_2_O_2_, HAuCl_4_, H_2_PtCl_6_, I_2_, Br_2_, and the disulfide DMS), and while our main focus is their reorientation behavior and the resulting swimming angles, we also report on swimming direction, velocity, relative efficiency, and underlying reaction mechanisms. By altering the material of the active cap or changing the fuel, we highlight the differences in these swimming systems, revealing a special reorientation phenomenon that remains consistent across the various microswimmer systems. When no fuel is added, Janus particles settle on the substrate with the heavier metal cap facing toward the substrate, showing an orthogonal alignment of their symmetric axis to the substrate. For all systems, after adding the fuel and initiating the reaction, the particles tend to reorient to approximately a 90° angle, resulting in the orientation where their symmetric axis is parallel to the substrate while they are active. In 2016, it was first reported that several factors influence the orientation of Janus particles, including bottom heaviness, solute distribution, hydrodynamic stress tensor, chemiosmotic contribution, and higher-order coupling.^6^ Through our experiments with different metal-capped particles exhibiting varying bottom heaviness values, we show that bottom heaviness does not significantly impact the reorientation process of the particles. Furthermore, by providing different reaction types using different fuel solutions of concentrations across a wide range from 0.015 to 1176 mM, we find that the density of solute distribution and molecular identity also have no major effect on the reorientation phenomenon. We conclude that the interplay between hydrodynamics, phoretic rotation, and chemiosmotic flow leads to a stable orientation at approximately 90°. The factors influencing the reorientation process can be restricted, given that cap density, chemical identity, and density differences were found to have a negligible impact. Demonstrated for a wide range of Janus particle materials and fuels, we show that the swimming angle is rather constant, and this orientation seems to favor effective propulsion.

The potential importance of osmotic slip in the behavior of chemically active colloids near walls received early recognition from Chiang and Velegol.^36^ Recently, evidence for the importance of this effect has been building from independent experimental efforts,^41,46–48^ especially from the careful statistical analysis of the motion of tracer particles.^48^ Here, we underscore the contribution of the chemiosmotic parameters to the reorientation process by conducting simulations with a continuum model for neutral self-diffusiophoresis. We vary the properties of the particles and the substrate to explore the influence of interactions with the formed products as well as the effective interactions with parameters *b*_w_, *b*_c_, and *b*_i_, in both inert and cap-forward swimming particles. This analysis reveals the effect of these properties on the theoretical angle distribution. For inert-forward particles, a minimally featured analytical model revealed the critical role of the hydrodynamic force quadrupole in biasing the sliding angle to be ≥ 90°. This hydrodynamic singularity may be less familiar than the force dipole, which registers the “pusher” or “puller” character of the propulsion mechanism, or the source dipole, which represents mass displacement by the finite size of the moving particle. It can be physically interpreted as representing the relative weakness of solute gradients (and therefore the interfacial slip velocity), as obtained in modeling studies,^49^ in the region of a particle “face” that is midway between the “equator” and “pole.” The significance of this term—previously recognized as critical in inducing oscillatory motion of squirmers in microchannels^50^—points to the subtle, but highly important, effects of the detailed distribution of the slip velocity on particle/wall interaction.^40,51^ Further work could focus on how this term and the other hydrodynamic singularities are affected by the propulsion mechanism (e.g., the distribution of catalytic activity^49,52^ ) and particle shape,^53,54^ with a view toward both understanding experimental results and deliberately engineering the properties of surface-bound sliding states.

## Materials and Methods

### Particle Preparation

The metal@SiO_2_ particles are produced using spherical 5 μm SiO_2_ beads sourced from Sigma-Aldrich, which are suspended in methanol. A monolayer of these beads is created by drop-casting the suspension onto 24 × 24 mm glass slides from VWR International. Beforehand, these slides are first cleaned with ethanol and then treated with oxygen plasma using electronic Diener plasma surface technology. To achieve a half-side metal coating on the SiO_2_ particles, a Quorum ES turbomolecular-pumped coater is employed for Pt deposition. Additionally, a thermal deposition device from Malz and Schmidt is used for coatings with copper and silver, enabling precise control over metal layer thickness. For experimental applications, the coated particles are suspended in water by transferring a part of the coated glass slide into an Eppendorf tube containing water. Ultrasonication is then applied to detach the particles from the glass surface.

### SEM and EDX Spectroscopy

For the SEM and EDX measurements, 100 μL of a prepared Janus particle suspension is placed in a 1.5 mL Eppendorf tube, and 100 μL of the assigned fuel is added. The mixture was shaken for several seconds and then left to react for approximately 1 h. Afterward, 2 μL of each sample is placed on an aluminum foil-coated sample holder and dried in air. The SEM measurements were taken using a Zeiss Gemini 300 instrument with an SE detector at 1.5 keV and a magnification of approximately 15,000x. The EDX measurements are performed using the same device and an Ultim Max 65 detector from Oxford Instruments Nanoanalysis, with varying voltages ranging from 6 to 12 eV, analyzed with the AZtec software from Oxford Instruments.

### X-ray Diffraction

To determine the products formed specifically for Ag@SiO_2_ particles in each halogen solution, a full slide of a particle monolayer is placed in an Eppendorf tube, and a 500 mM solution of either I_2_ or Br_2_ is added. The mixture is shaken for a few seconds and allowed to react for approximately 1 h. After the reaction, the sample is collected by centrifuging at 10000 rpm for 2 min. The sediment at the bottom of the tube is then used to place several drops onto an XRD sample holder, which is dried in air. X-ray diffraction (XRD) is performed over a 2θ range of 10–90° for 2.5 h using a D2 PHASER device from Bruker.

### Angle and Velocity Measurements

For all microscopy measurements, an Axio Observer optical microscope from Carl Zeiss Microscopy GmbH, equipped with an Axiocam 702 Mono and a Colibri 7 light source, was utilized. All videos were captured using a 50× magnification at 40 frames per second. To collect the videos, ethanol and plasma-cleaned 24 × 24 mm glass slides from VWR International were modified with imaging spacers from Grace Bio-Lab’s SecureSeal to define a specific reaction area. Next, 5 μL of the well-ultrasonically suspended and freshly prepared particle suspension was placed on the glass substrate. For the H_2_O_2_ fuel solution, 20 μL of various concentrated liquids were added to achieve the final concentrations displayed in Figure 2. For the other fuel systems, 5 μL of fuel was added to the 5 μL particle suspension to obtain the desired final concentrations. After adding the fuel, the video was recorded 5 s later to ensure the reaction took place.

For tracking, velocity measurements, and MSD calculation, MATLAB R2022a software is used. The code tracks the particle’s location using a binary video to identify particles within a specified area, diameter, and shape. After the location tracking, it calculates the velocity using the change in position over time. The mean velocity at different concentrations is then calculated as the average velocity of each tracked particle in the first 600 frames. Particles attached to the substrate are not considered for tracking and calculations. Additionally, the mean squared displacement is calculated by measuring the difference between two positions at different time steps and then squaring that value.^55^ Following the averages of these squared displacements across all available time intervals determines the mean squared displacement.

To determine the angles, the same videos as for velocity measurement are used. First, they are transformed into binary ones by setting a threshold called the BW value. As shown in Figure S3b, the cap of the particle appears as a white area, while the border of the particle forms a thin white line. To focus solely on the cap in the binary image and eliminate the border of the particle, a morphological opening operation is utilized using the “imopen” function. This function removes the border on the inert side of the particle without losing any pixels from the cap. Consequently, the resulting frames display only the metal cap, as shown in Figure S3c. Based on this, the area of the white space can be calculated for each particle in each frame of the video, generating the value of *A*_c_. To calculate the angle of the particle, the area of the entire particle is also needed. Therefore, the “minboundcircle” function^56^ is employed to generate a circle that fits around the white area, resulting in theoretically fitted circles, as shown in Figure S3d. From this, the area of each circle can be calculated for each particle in every frame, providing the variable *A*_0_, using eq 3.

$$\theta =\cos\left(\frac{A_{0}}{\pi \cdot {\left(\frac{A_{c}}{2}\right)}^{2}}-1\right)$$ 3

To validate this method, reference measurements were conducted using Cu@Ni@SiO_2_ particles, which were manipulated by a magnetic field to achieve a specific orientation. The results, presented in Figure S4, demonstrate good alignment between the set and the determined angles, confirming the applicability of this method for this project. Furthermore, the impact of the set BW value threshold was analyzed in Figure S5. This analysis indicates that within a range of ±20, the effect on angle determination is minimal. However, there comes a point where the border of the particle on the inner side can no longer be effectively removed using the “imopen” function, resulting in the particle being recognized fully rather than just its cap. This leads to angle values approaching zero.

### Theoretical Model

We adapt a widely used model for a particle that moves near a solid planar wall by neutral diffusiophoresis.^34^ Specifically, the a spherical particle of radius *R* is located at position **x**_p_ = (*x*_p_, *y*_p_, *z*_p_ = *h*) in a stationary Cartesian reference frame, and a solid planar wall is located at *z* = 0. The particle emits a reaction product (“solute”) at a constant and uniform rate κ over a hemispherical catalytic cap. Assuming small Péclet number (negligible advection), the concentration (number density) of solute *c*(**x**) is governed by the Laplace equation, ∇^2^ *c* = 0. The concentration field is subject to the boundary conditions ∇*c*·**n** **^** = 0 on the wall (*z* = 0) and on the inert “face” of the particle, and −*D*[∇*c*·**n** **^**] = κ on the catalytic “face,” where *D* is the diffusion coefficient of the solute. Additionally, the solute concentration vanishes far from the particle, i.e., *c*(**x**) → 0 as |**x**|→*∞*.

The particle is immersed in an incompressible Newtonian fluid with dynamic viscosity μ. Assuming small Reynolds number, the fluid velocity **u**(**x**) is governed by the Stokes equation −∇*p* + μ∇^2^ **u** = 0 and the incompressibility condition ∇·**u** = 0, where *p*(**x**) is the pressure. The velocity is subject to boundary conditions **u**(**x**_s_) = **v**_s_(**x**_s_) on the planar wall and **u**(**x**_s_) = **U** + **Ω** × (**x**_s_ – **x**_p_) + **v**_s_(**x**_s_) on the particle surface, where **x**_s_ is a position at a fluid/solid interface, and **U** and **Ω** are, respectively, the unknown translational and angular velocities of the particle. The quantity **v**_s_(**x**_s_) is the interfacial slip velocity and is obtained from the solution for *c*(**x**) as **v**_s_(**x**_s_) = −*b*(**x**_s_)∇_||_ *c*. Here, *b*(**x**_s_) is the so-called surface mobility or phoretic mobility (discussed below), and $\nabla_{\left|\right|}=(I-\hat{n}\hat{n})$ is the surface gradient operator. Additionally, the fluid velocity vanishes far from the particle, i.e., |**u**(**x**)|→ 0 as |**x**|→*∞*. Assuming vanishing Reynolds number, the all forces and torques on the particle instantaneously balance: **F**_h_ + **F**_g_ + **F**_ev_ = 0 and **τ**_h_ + **τ**_g_ + **τ**_ev_ = 0. Here, the hydrodynamic force is **F**_h_ = ∫_*S*_p__ **σ**·**n** d*S* and the hydrodynamic torque is **τ**_h_ = ∫_*S*_p__(**x**_s_ + **x**_p_) × **σ**·**n** d*S*, where the Newton stress tensor is $\sigma =-pI+\mu \left(\nabla u+\nabla u^{T}\right)$ . The integrals are taken over the surface of the particle, denoted by *S*_p_. The gravitational and excluded volume forces and torques are discussed below.

As noted in the main text, we take *b* to be piecewise constant over the bounding solid surfaces (wall and particle), with *b* = *b*_w_ *b*_0_ on the wall, *b* = *b*_c_ *b*_0_ on the catalytic face of the particle, and *b* = *b*_i_ *b*_0_ on the inert face of the particle. The quantities *b*_i_, *b*_c_, and *b*_w_ are dimensionless parameters, and *b*_0_ is a characteristic phoretic mobility with units of m^5^ s^–1^. The value of *b*_0_ determines the characteristic swimming velocity *U*_0_ ≡ *b*_0_κ/*D*, which is used to nondimensionalize the gravitational and excluded volume forces (see below).

The linearity of the Stokes equation permits the contributions of activity, gravity, and excluded volume to be calculated separately and superposed, giving **U** = **U**_a_ + **U**_g_ + **U**_ev_ and **Ω** = **Ω**_a_ + **Ω**_g_ + **Ω**_ev_. The contributions from activity, **U**_a_ and **Ω**_a_, can themselves be split into contributions from the diffusiophoretic slip on the particle surface and the diffusioosmotic slip on the planar wall: **U**_a_ = **U**_dp_ + **U**_ws_ and **Ω**_a_ = **Ω**_dp_ + **Ω**_ws_. We calculate these quantities using the Lorentz Reciprocal Theorem (LRT), as detailed in ref 6. Specifically, for a given instantaneous height and orientation of the particle, application of the LRT gives

$$R\cdot \left(\begin{array}{l} U_{a}\\ \Omega_{a} \end{array}\right)=b$$ 4

where the six-component right-hand side vector **b** is

$$b_{j}=\int_{S}v_{s}(x_{s})\cdot \sigma^{\left(j\right)}\cdot \hat{n}dS$$ 5

where the integral is taken over the surface of the particle and the wall. The quantity R is the 6 × 6 hydrodynamic resistance matrix for a sphere at height *h* above a no-slip wall. The *i*-th column of R is constructed as (**F**_H_, **τ**_H_)^T^ from the hydrodynamic force **F**_H_ and torque **τ**_H_ for a particle either translating with unit velocity in the *x*, *y*, or *z* direction, corresponding to *i* = 1, *i* = 2, and *i* = 3, respectively, or rotating with unit angular velocity in the *x*, *y*, or *z* direction, corresponding to *i* = 4, *i* = 5, and *i* = 6. Likewise, the index *j* ∈ {1, 2, ···, 6} specifies one of these modes of motion, and **σ** ^(*j*)^ is the hydrodynamic stress obtained for that mode.

One advantage of the LRT approach is that it allows for decomposition of chemi-osmotic (wall slip) and self-diffusiophoretic (particle slip) contributions to the rotation of a chemically active particle near a wall. Additionally, the self-diffusiophoretic rotation can be further decomposed into hydrodynamic and phoretic contributions, i.e., contributions due to confinement by the wall of the flow and chemical fields, respectively. To isolate the wall slip contribution **Ω**_ws_, we evaluate the integral in eq 5 only over the wall. Likewise, to isolate the self-diffusiophoretic contribution **Ω**_dp_, we evaluate the integral in eq 5 only over the particle surface. Additional analysis is needed to decompose **Ω**_dp_ into hydrodynamic and phoretic contributions.^6^ Specifically, we write **σ** ^(*j*)^ = **σ**_fs_^(*j*)^ + δ**σ** ^(*j*)^, $R=R_{\mathrm{fs}}+\delta R$ , and **v**_s_(**x**_s_) = **v**_s,fs_(**x**_s_) + δ**v**_s_(**x**_s_). Here, the subscript “fs” indicates values obtained for a particle in free space, i.e., far away from the wall, and the quantities with δ are modifications induced by the presence of the wall. To isolate the hydrodynamic contribution **Ω**_hi_ to particle rotation, we use **σ** ^(*j*)^, R , and **v**_s,fs_(**x**_s_) in eqs 4 and 5, and evaluate the integral over the surface of the particle (not the wall). To isolate the phoretic contribution **Ω**_pr_, we use **σ**_fs_^(*j*)^, $R_{\mathrm{fs}}$ , and δ**v**_s_(**x**_s_) in eqs 4 and 5, and again evaluate the integral over the particle surface. We note that the decomposition of the phoretic and hydrodynamic contributions neglects higher-order coupling between the hydrodynamic and chemical confinements, i.e., contributions to particle rotation involving two or more δ terms. Accordingly, **Ω**_dp_ ≠ **Ω**_pr_ + **Ω**_hi_. However, in numerical calculations, we find that |**Ω**_dp_ – **Ω**_pr_ – **Ω**_hi_| is small.^6^

In order to confine the particle near the wall, we include a gravitational force **F**_g_ = −*GF*_0_ **z** **^**, where *G* is a dimensionless parameter, and *F*_0_ = μ*RU*_0_ is a characteristic force. We calculate the contribution of **F**_g_ to the particle’s generalized velocity V=(U,Ω) as $V_{g}=R^{-1}F_{g}$ , where $V_{g}=(U_{g},\Omega_{g})$ and $F_{g}=(F_{g},\tau_{g})$ . We neglect bottom heaviness, so that **τ**_g_ = **0**. For “heavy” particles, we choose *G* = 6π, such that the dimensionless sedimentation velocity of the particle is approximately unity when the particle is far from the wall, i.e., |**U**_g_|/*U*_0_ ≈ 1 when *h*/*R* ≫ 1. Since, for surface mobility parameters considered here, the active particle always has a dimensionless propulsion velocity less than unity, the gravitational force confines the particle to the vicinity of the wall.

In order to prevent the particle from “crashing” into the wall, we include the following short-range excluded volume force:

$$F_{\mathrm{ev}}=EF_{0}\frac{\tau R\exp(-\tau \delta )}{1-\exp(-\tau \delta )}\hat{z}$$ 6

where *E* is a dimensionless quantity, τ^–1^ is the length scale of the repulsive force, and δ = *h* – *R* is the particle/wall gap distance. To make the excluded volume force short-ranged, we choose *τR* = 100. We also choose *E* = 10. The contribution of **F**_ev_ to the generalized velocity $V_{\mathrm{ev}}=(U_{\mathrm{ev}},\Omega_{\mathrm{ev}})$ is calculated as $V_{\mathrm{ev}}=R^{-1}F_{\mathrm{ev}}$ , where $F_{\mathrm{ev}}=(F_{\mathrm{ev}},0)$ . There is no excluded volume torque: **τ**_ev_ = 0.

The concentration field *c*(**x**), hydrodynamic resistance matrix R , and stress tensor **σ** ^(*j*)^ are evaluated numerically with the BEM,^17^ and the integrals within eq 5 are evaluated by quadrature. In order to scan the space of (*b*_i_, *b*_w_) or (*b*_c_, *b*_w_) for the sliding angle, we numerically integrate particle trajectories that start from an initial height *h*/R = 1.05 and angle θ = 90° for a dimensionless time *T*/*T*_0_ = 10,000. We use Euler integration with a dimensionless time step δ*t*/*T*_0_ = 0.025. The final height and angle of the particle are recorded; if the final height is *h*/*R* ≥ 6, the particle is considered to have left (scattered from) the wall. While this automated method makes consideration of a large parameter space tractable, it has some limitations. It can miss an attractor if the initial state is not in the attractor’s basin of attraction—although the starting angle and height are chosen so as to be near any θ ≈ 90° attractor. By construction, bistability (the presence of two attractor states) is not detected. Moreover, under some circumstances, the excluded volume potential can lead to limit cycles (i.e., sustained oscillations in the height and angle of the particle). The automated method will record only one arbitrary point in the limit cycle. Therefore, to test the fidelity of the method, we compare the data set in Figure 6a against a data set calculated “manually,” i.e., by laborious human inspection of phase portraits for each parameter set (see Figure S22). We find excellent quantitative agreement between the two figures, although Figure S22 resolves the mean angle of two points that have limit cycles (square symbols), and bistability at one point (circle with two colors). We conclude that the automated method reliably finds attractors, especially the attractors of interest with θ ≈ 90°.

## Supplementary Material















## Notes

Experimental results and characterization data are available: a table of densities and absolute mass contributions (Table S1); MSD curves for Janus particles in the passive state (Figure S1); reorientation angle over time for Janus particles in the passive state (Figure S2); visualization of the angle determination (Figure S3); reference angle measurements (Figure S4); influence of the BW value (Figure S5); detailed SEM analysis of Janus particles after reaction with H_2_O_2_ (Figure S6); MSD curves for Janus particles in different concentrations of H_2_O_2_ (Figure S7); reorientation angle over time for Janus particles in different concentrations of H_2_O_2_ (Figure S8); results for the reaction of Ag@SiO_2_ with noble-metal acids, including a schematic overview of the mechanism, SEM and EDX measurements, and angle and velocity data (Figure S9); MSD curves for Ag@SiO_2_ and Cu@SiO_2_ with different concentrations of noble-metal acids (Figure S10); angle stability over time for Ag@SiO_2_ and Cu@SiO_2_ with different concentrations of noble-metal acids (Figure S11); XRD data for AgI and AgBr (Figure S12); MSD curves for Ag@SiO_2_ and Cu@SiO_2_ with different concentrations of halogens (Figure S13); angle stability over time for Ag@SiO_2_ and Cu@SiO_2_ with different concentrations of halogens (Figure S14); MSD curves for Cu@SiO_2_ with different concentrations of sulfides (Figure S15); and angle stability over time for Cu@SiO_2_ with different concentrations of sulfides (Figure S16); Sliding angles for a particle with *b*_c_ = −1 for the case of a neutrally buoyant particle (left) and a “heavy” particle (right) (Figure S17); Sliding angles for a particle with *b*_i_ = −1 for the case of a neutrally buoyant particle (left) and a “heavy” particle (right). Diamond symbols indicate sliding states with motion reversal (Figure S18); Sliding angles for a particle with *b*_c_ = 1 for the case of a neutrally buoyant particle (left) and a “heavy” particle (right) (Figure S19); Sliding angles for a particle with *b*_i_ = 1 for the case of a neutrally buoyant particle (left) and a “heavy” particle (right). Diamond symbols indicate sliding states with motion reversal (Figure S20); (left) Sliding angles in the analytical model for *b*_c_ = −1 and with the force quadrupole term omitted (γ_HI_ = 0) as a function of bw and bi. The white line shows the contour with θ = 90°. (right) Histogram showing the distribution of sliding angles for angles in the range 5° < θ < 175°. The distribution is approximately symmetric with respect to 90° (Figure S21); Sliding angles for a particle with *b*_c_ = −1 for the case of a neutrally buoyant particle. These sliding angles were determined by manual inspection of individual phase portraits. Square symbols indicate limit cycles, with the color showing the cycle-averaged angle. The one symbol with two colors indicates bistability (two coexisting stable attractors) (Figure S22); detailed explanation of determination of prefactors in analytical model (PDF)
Video S1 - Long-duration video of Pt@SiO_2_ in 1176 mM H_2_O_2_ (AVI)
Video S2 - Pt@SiO_2_, Ag@SiO_2_ and Cu@SiO_2_ in different concentrated H_2_O (AVI)
Video S3 - Ag@SiO_2_ and Cu@SiO_2_ in different concentrated noble metal acids (HAuCl_4_, H_2_PtCl_6_) (AVI)
Video S4 - Ag@SiO_2_ and Cu@SiO_2_ in different concentrated halogens (I_2_, Br_2_) (AVI)
Video S5 - Cu@SiO2 in different concentrated DMS (AVI)
Video S6 - Pt@SiO_2_, Ag@SiO_2_, and Cu@SiO_2_ without fuel (passive system) (AVI)
