# Supplementary material for: Understanding Universal Reorientation Behavior of Active Janus Colloids
Source: ACS Nano. 2026 Jul 27;20(31):21708–22. doi: 10.1021/acsnano.6c03119 (PMC13472329; doi:10.1021/acsnano.6c03119)
Supplement: Supplementary file 1 [file nn6c03119_si_001.pdf]

Supporting Information - Understanding Universal Reorientation Behaviour of Active Janus Colloids

Kelly Henze,*,† William E. Uspal,*,‡ Martin Wittmann,*,† and Juliane Simmchen*,†,¶

†*Physical Chemistry, TU Dresden, Dresden*

‡*Department of Mechanical Engineering, University of Hawaii, Mānoa*

¶*Pure and Applied Chemistry, University of Strathclyde, Glasgow*

E-mail: kelly.henze@tu-dresden.de; uspal@hawaii.edu; martin.wittmann@tu-dresden.de; juliane.simmchen@strath.ac.uk

- PDF - Additional experimental results and characterization data are available: a table of densities and absolute mass contributions (Table S1); MSD curves for Janus particles in the passive state (Figure S1); reorientation angle over time for Janus particles in the passive state (Figure S2); visualization of the angle determination (Figure S3); reference angle measurements (Figure S4); influence of the BW value (Figure S5); detailed SEM analysis of Janus particles after reaction with H_2O_2 (Figure S6); MSD curves for Janus particles in different concentrations of H_2O_2 (Figure S7); reorientation angle over time for Janus particles in different concentrations of H_2O_2 (Figure S8); results for the reaction of Ag@SiO_2 with noble metal acids, including a schematic overview of the mechanism, SEM and EDX measurements, and angle and velocity data (Figure S9); MSD curves for Ag@SiO_2 and Cu@SiO_2 with different concentrations of noble

metal acids (Figure S10); angle stability over time for $Ag@SiO_2$ and $Cu@SiO_2$ with different concentrations of noble metal acids (Figure S11); XRD data for AgI and AgBr (Figure S12); MSD curves for $Ag@SiO_2$ and $Cu@SiO_2$ with different concentrations of halogens (Figure S13); angle stability over time for $Ag@SiO_2$ and $Cu@SiO_2$ with different concentrations of halogens (Figure S14); MSD curves for $Cu@SiO_2$ with different concentrations of DMS (Figure S15); and angle stability over time for $Cu@SiO_2$ with different concentrations of DMS (Figure S16); Sliding angles for a particle with $\mathrm{b_c} = -1$ for the case of a neutrally buoyant particle (left) and a "heavy" particle (right) (Figure S17); Sliding angles for a particle with $\mathrm{b_i} = -1$ for the case of a neutrally buoyant particle (left) and a "heavy" particle (right). Diamond symbols indicate sliding states with motion reversal (Figure S18); Sliding angles for a particle with $\mathrm{b_c} = 1$ for the case of a neutrally buoyant particle (left) and a "heavy" particle (right) (Figure S19); Sliding angles for a particle with $\mathrm{b_i} = 1$ for the case of a neutrally buoyant particle (left) and a "heavy" particle (right). Diamond symbols indicate sliding states with motion reversal (Figure S20); (left) Sliding angles in the analytical model for $\mathrm{b_c} = -1$ and with the force quadrupole term omitted ($\gamma_{\mathrm{HI}} = 0$) as a function of $\mathrm{b_w}$ and $\mathrm{b_i}$. The white line shows the contour with $\theta = 90°$. (right) Histogram showing the distribution of sliding angles for angles in the range $5° < \theta < 175°$. The distribution is approximately symmetric with respect to 90° (Figure S21); Sliding angles for a particle with $\mathrm{b_c} = -1$ for the case of a neutrally buoyant particle. These sliding angles were determined by manual inspection of individual phase portraits. Square symbols indicate limit cycles, with the color showing the cycle-averaged angle. The one symbol with two colors indicates bistablity (two co-existing stable attractors) (Figure S22); detailed explanation of determination of prefactors in analytical model.

- Video S1 - Long duration video of $Pt@SiO_2$ in 1176 mM H_2O_2
- Video S2 - $Pt@SiO_2$, $Ag@SiO_2$ and $Cu@SiO_2$ in different concentrated H_2O_2

- Video S3 - Ag@SiO_2 and Cu@SiO_2 in different concentrated noble metal acids ($HAuCl_4$, H_2PtCl_6)
- Video S4 - Ag@SiO_2 and Cu@SiO_2 in different concentrated halogens (I_2, Br_2)
- Video S5 - Cu@SiO2 in different concentrated DMS
- Video S6 - Pt@SiO_2, Ag@SiO_2, and Cu@SiO_2 without fuel (passive system)

Table S1: Overview of densities and absolute mass contribution of the metal caps and SiO_2 to the whole Janus particle.

Material	Density ($g \cdot cm^{-3}$)	Cap Thickness (nm)	Volume (cm^3)	Mass (g)
SiO_2	2.65 [1]	-	6.55×10^{-11}	1.72×10^{-10}
Pt	21.45 [1]	30	1.19×10^{-12}	2.56×10^{-11}
Ag	10.5 [1]	30	1.19×10^{-12}	1.25×10^{-11}
Cu	8.96 [1]	30	1.19×10^{-12}	1.07×10^{-11}

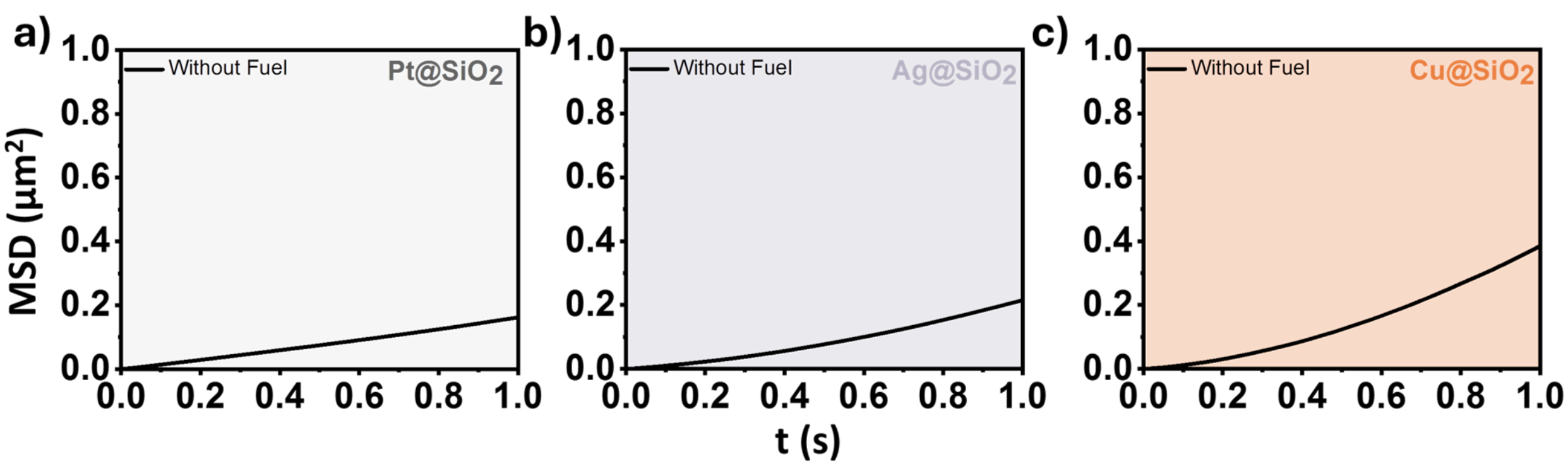

Figure S1: MSD curves for Pt@SiO_2, Ag@SiO_2, and Cu@SiO_2 in their passive states without any fuel addition.

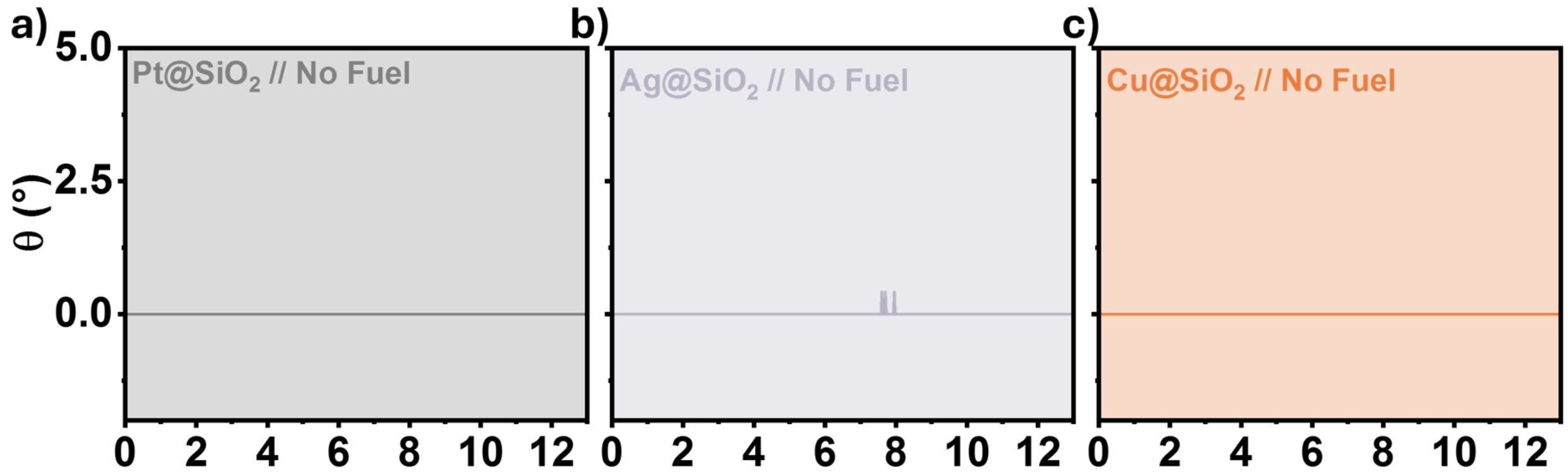

Figure S2: Reorientation angle over time for Pt@SiO_2, Ag@SiO_2, and Cu@SiO_2 in their passive states without any fuel addition.

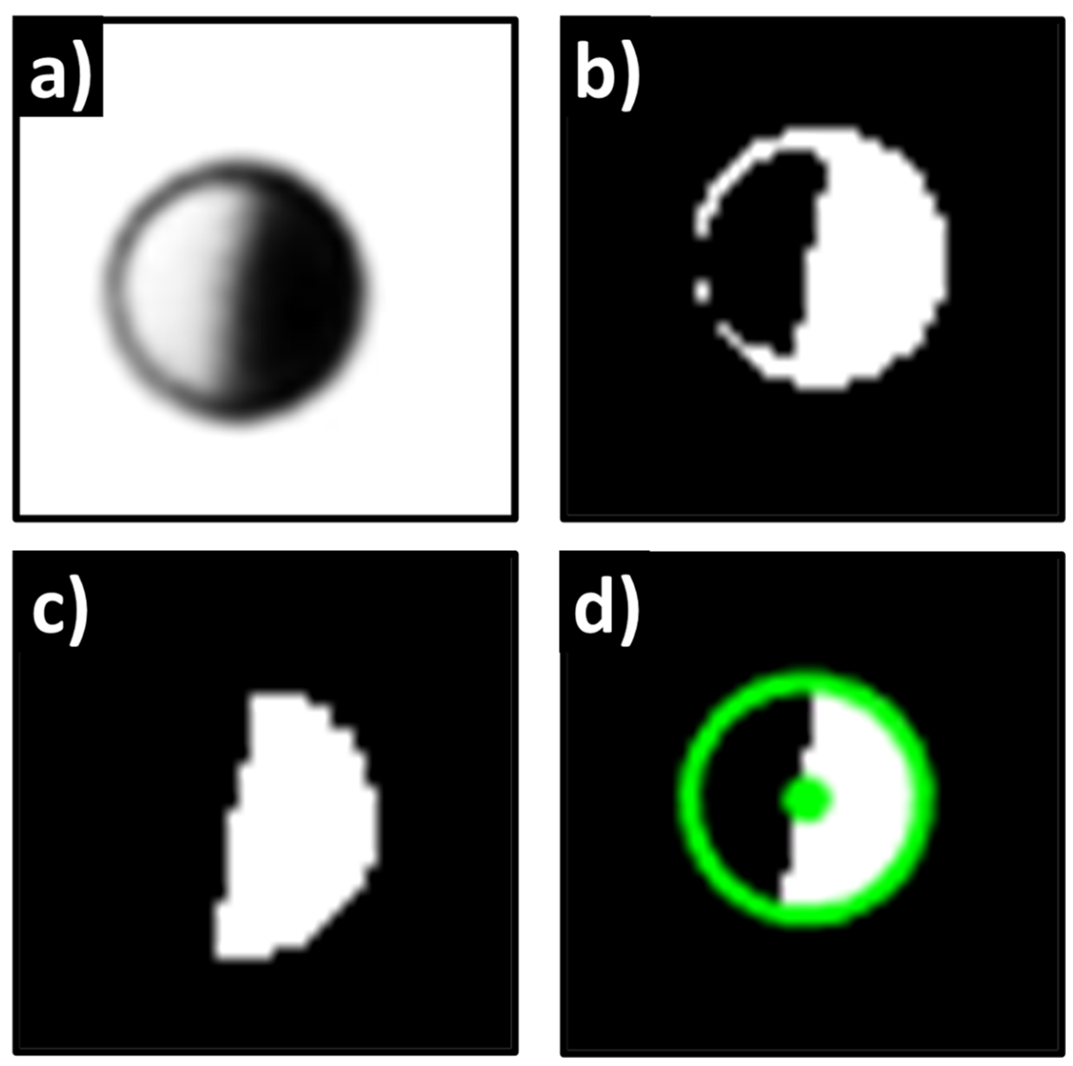

Figure S3: Angle determination process with a) plain microscopy image, b) binarised image, c) binarised image after morphological opening, d) binarised image after morphological opening with the minimum bound circle (green).

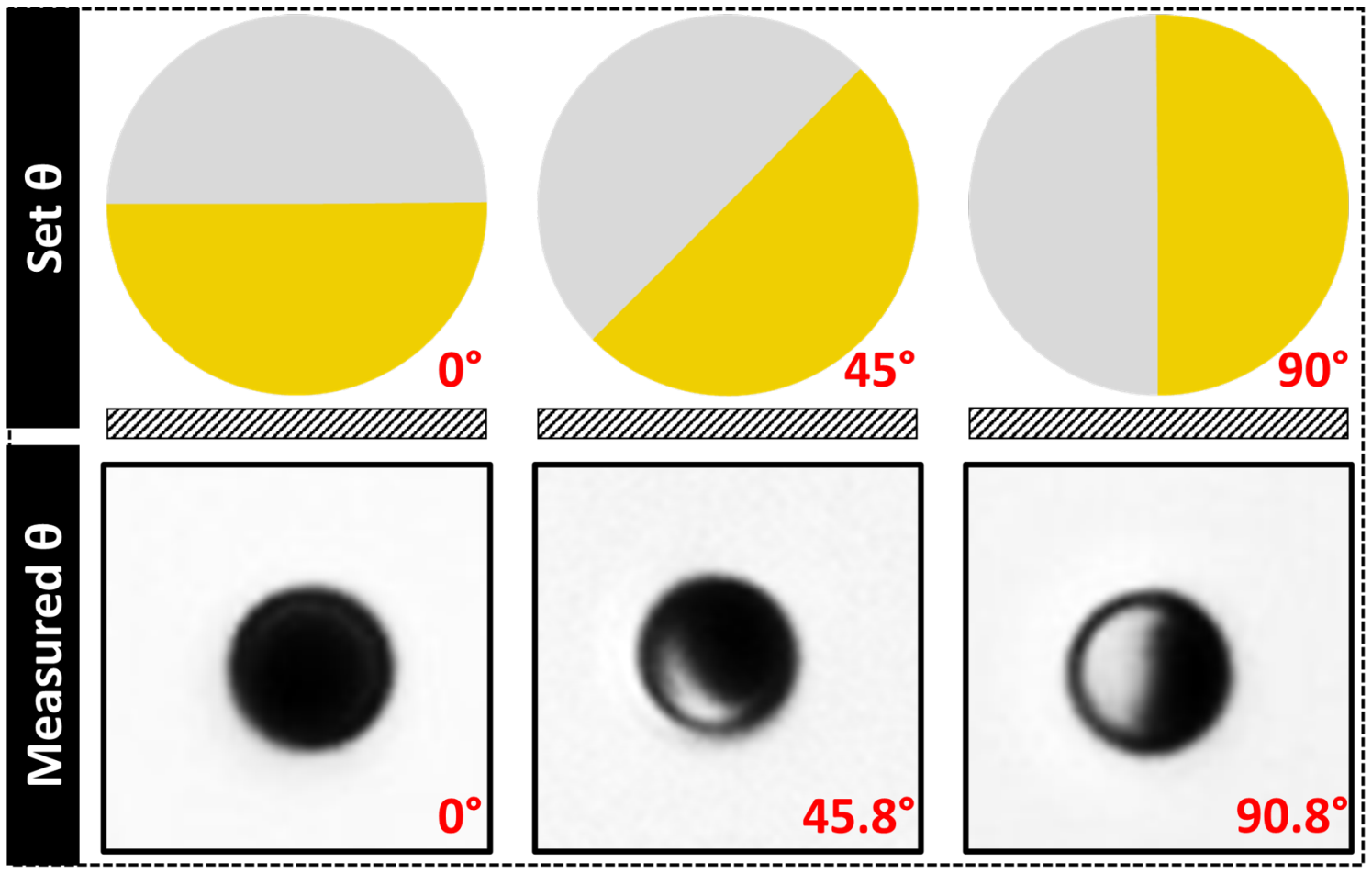

Figure S4: Angle determination - referenece measurements for 0°, 45° and 90°

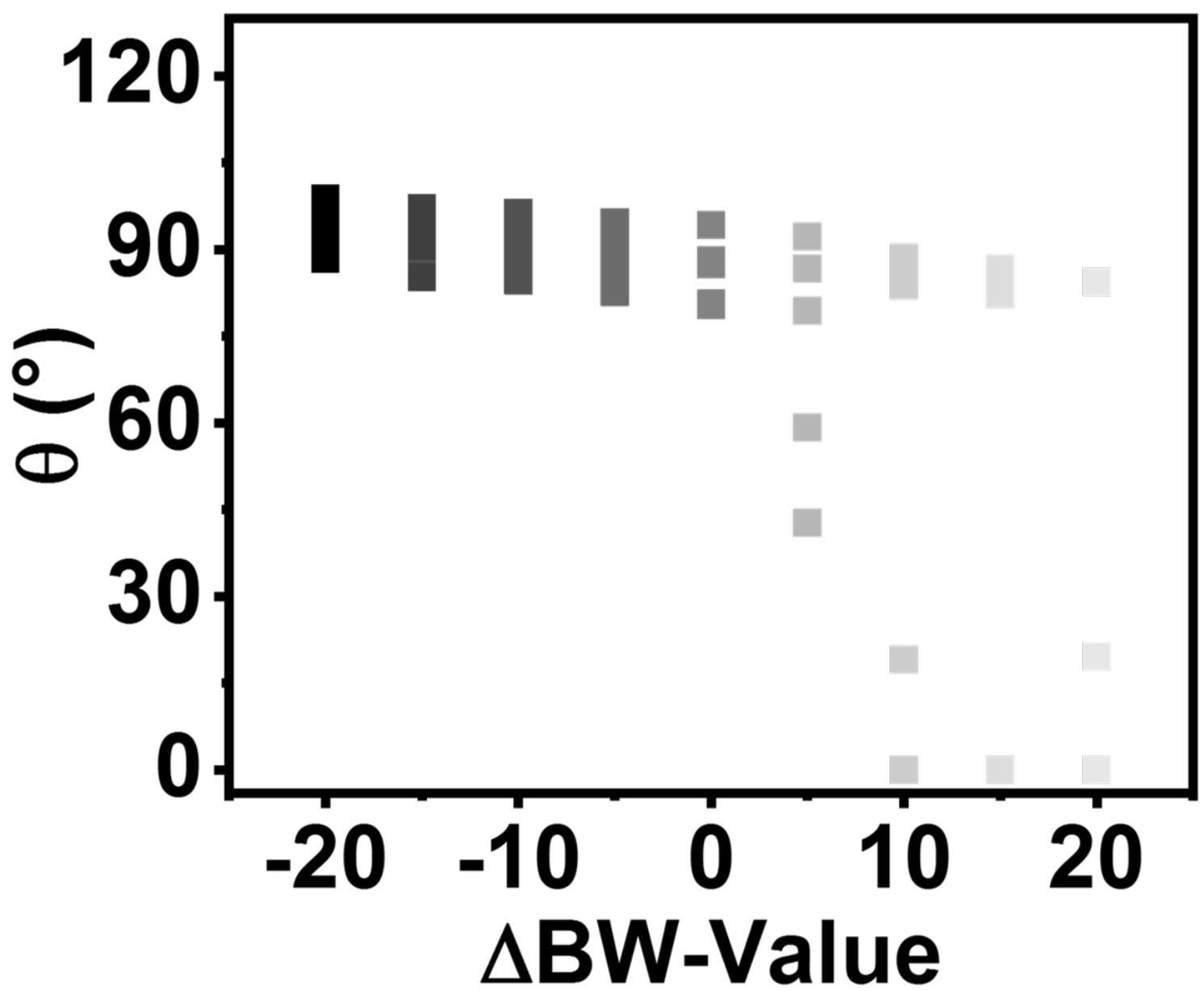

Figure S5: Influence of BW-Values setting to reorientation angle (θ)

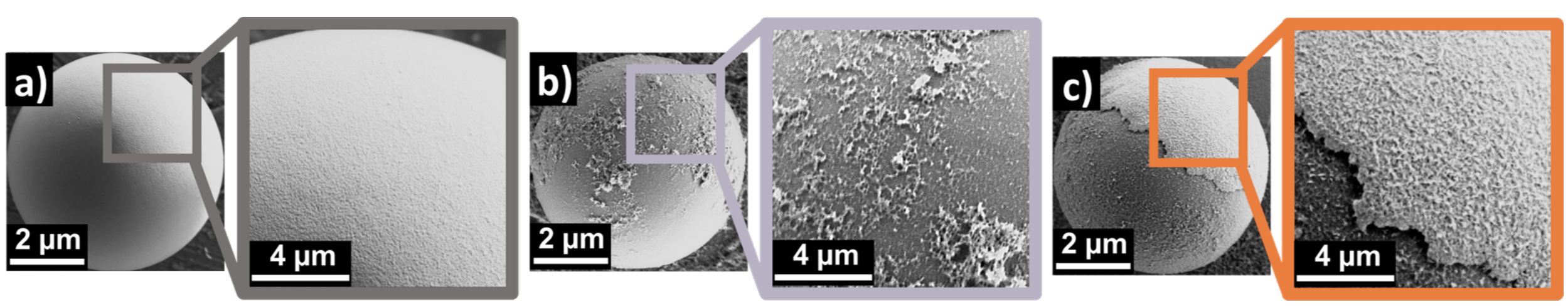

Figure S6: SEM analysis of Pt@SiO_2, Ag@SiO_2, and Cu@SiO_2 after the addition of 1176 mM H_2O_2 and approximately 1 h reaction time, including close-up images of the metal cap.

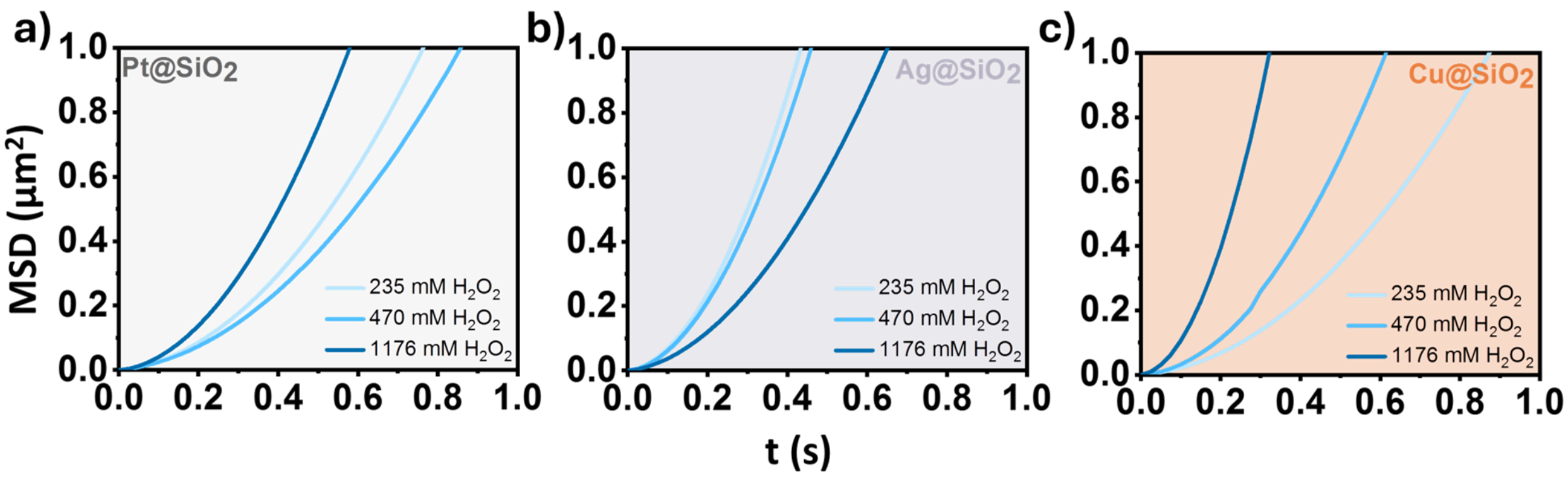

Figure S7: MSD curves for Pt@SiO_2, Ag@SiO_2, and Cu@SiO_2 while reacting with H_2O_2 at different concentrations of 235, 470, and 1176 mM.

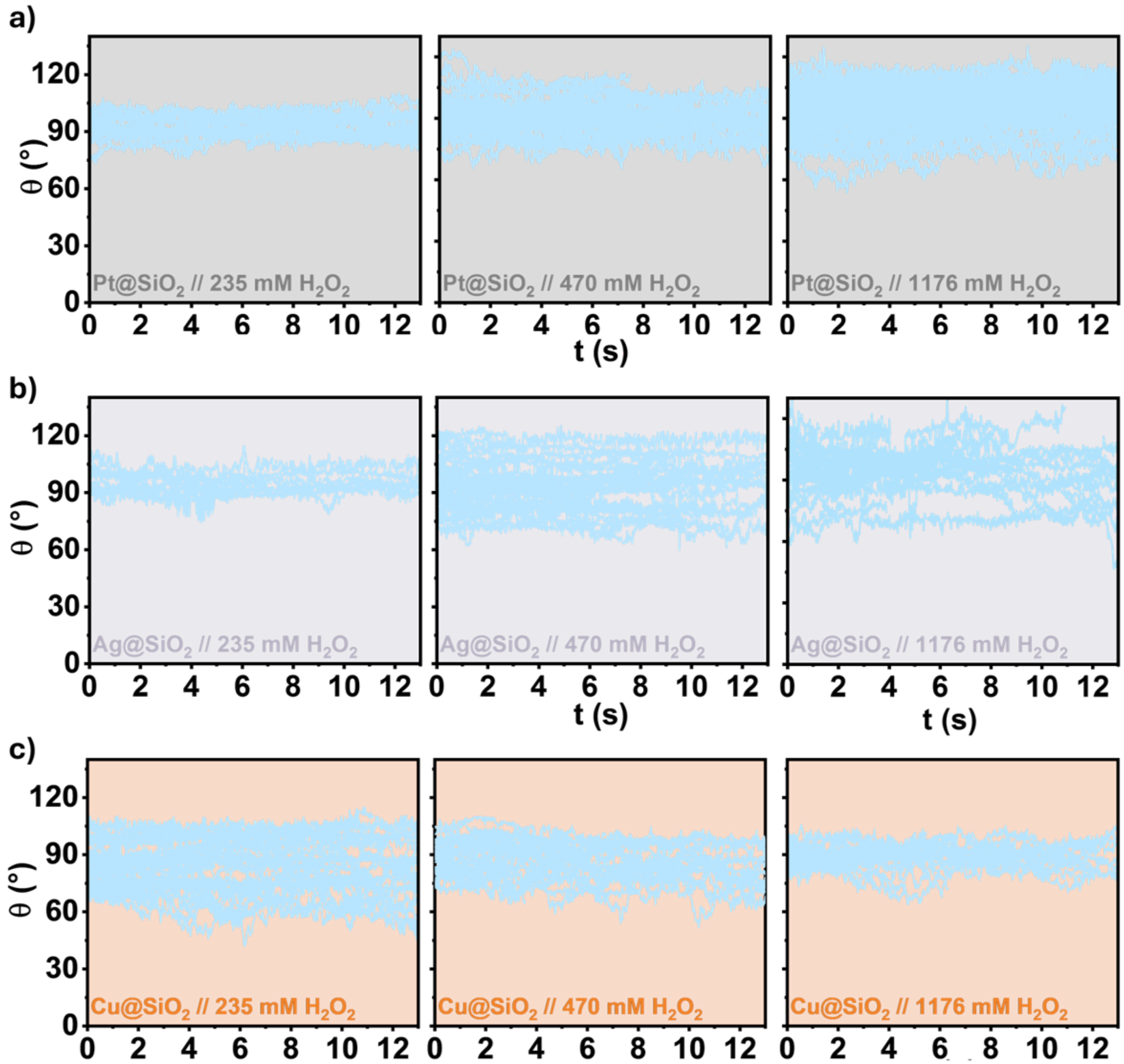

Figure S8: Reorientation angle over time for $Pt@SiO_2$, $Ag@SiO_2$, and $Cu@SiO_2$ while reacting with H_2O_2 at different concentrations of 235, 470, and 1176 mM.

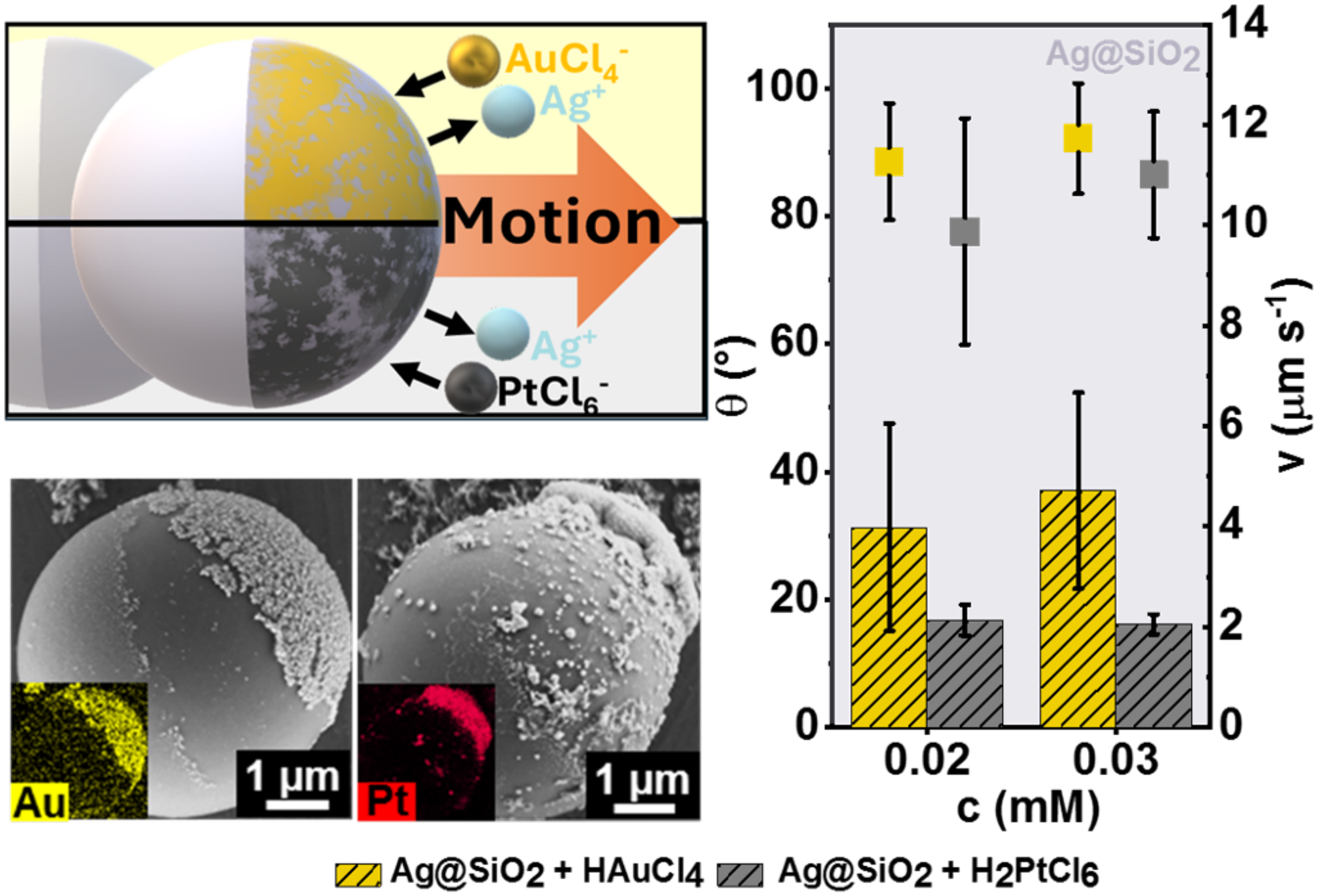

Figure S9: Results for the reaction of $AG@SiO_2$ with noble metal acids such as $HAuCl_4$ and H_2PtCl_6 include a schematic overview of the mechanism, as well as SEM and EDX measurement results. Additionally, the dependency of the reorientation angle and velocity on varying concentrations of the noble metal acid fuels is displayed.

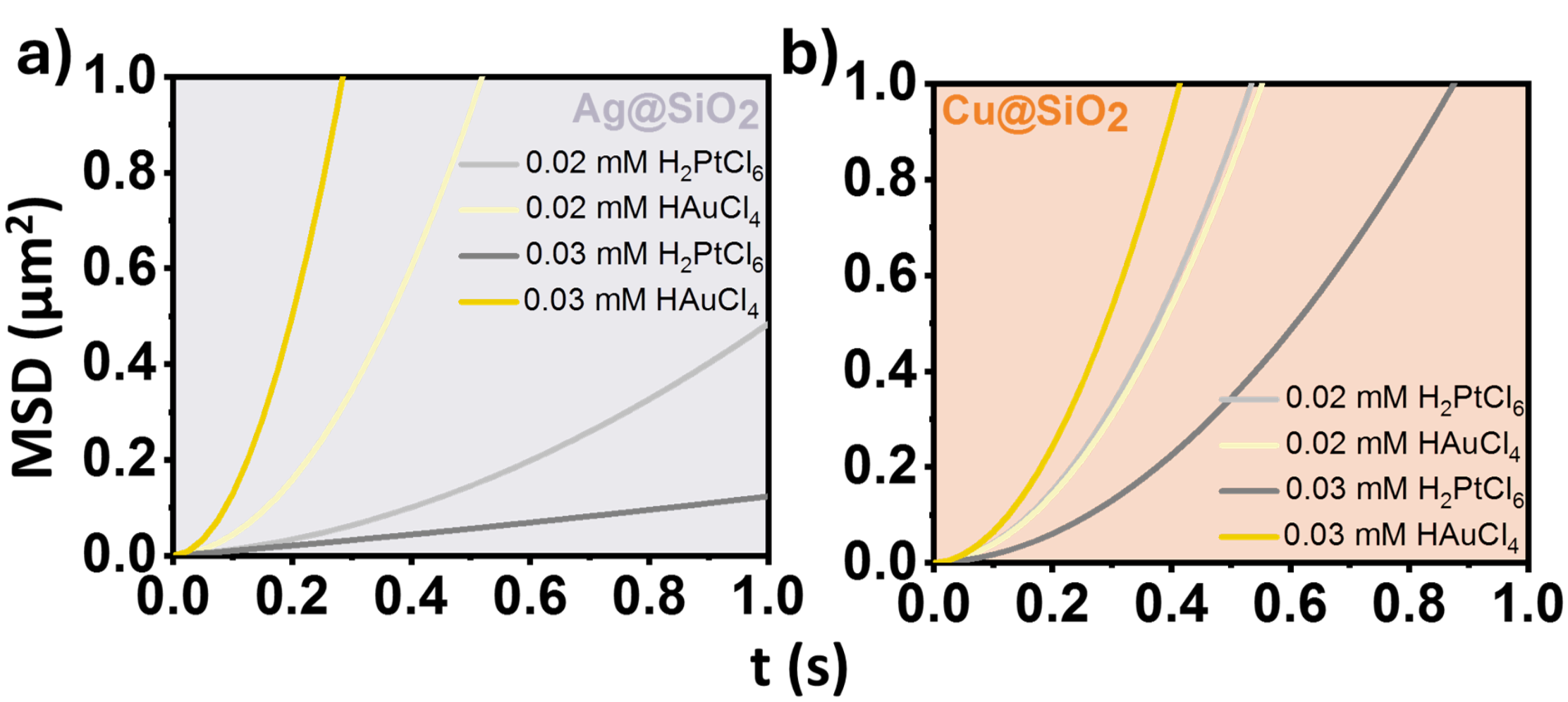

Figure S10: MSD curves for $Ag@SiO_2$, and $Cu@SiO_2$ while reacting with noble metal acids such as $HAuCl_4$ and H_2PtCl_6 at different concentrations of 0.02 and 0.03 mM.

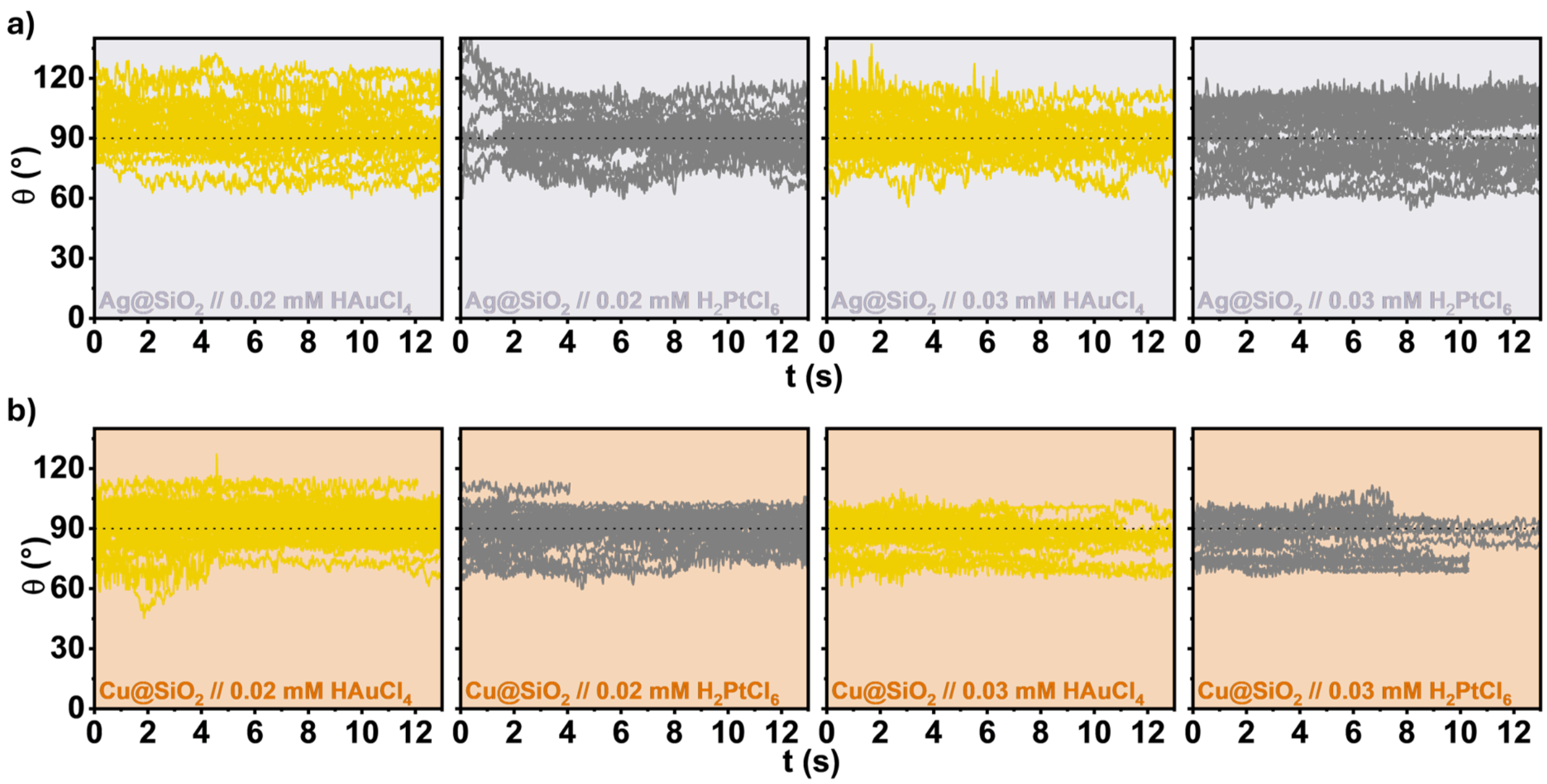

Figure S11: Reorientation angle over time for Ag@SiO_2, and Cu@SiO_2 while reacting with noble metal acids such as $HAuCl_4$ and H_2PtCl_6 at different concentrations of 0.02 and 0.03 mM.

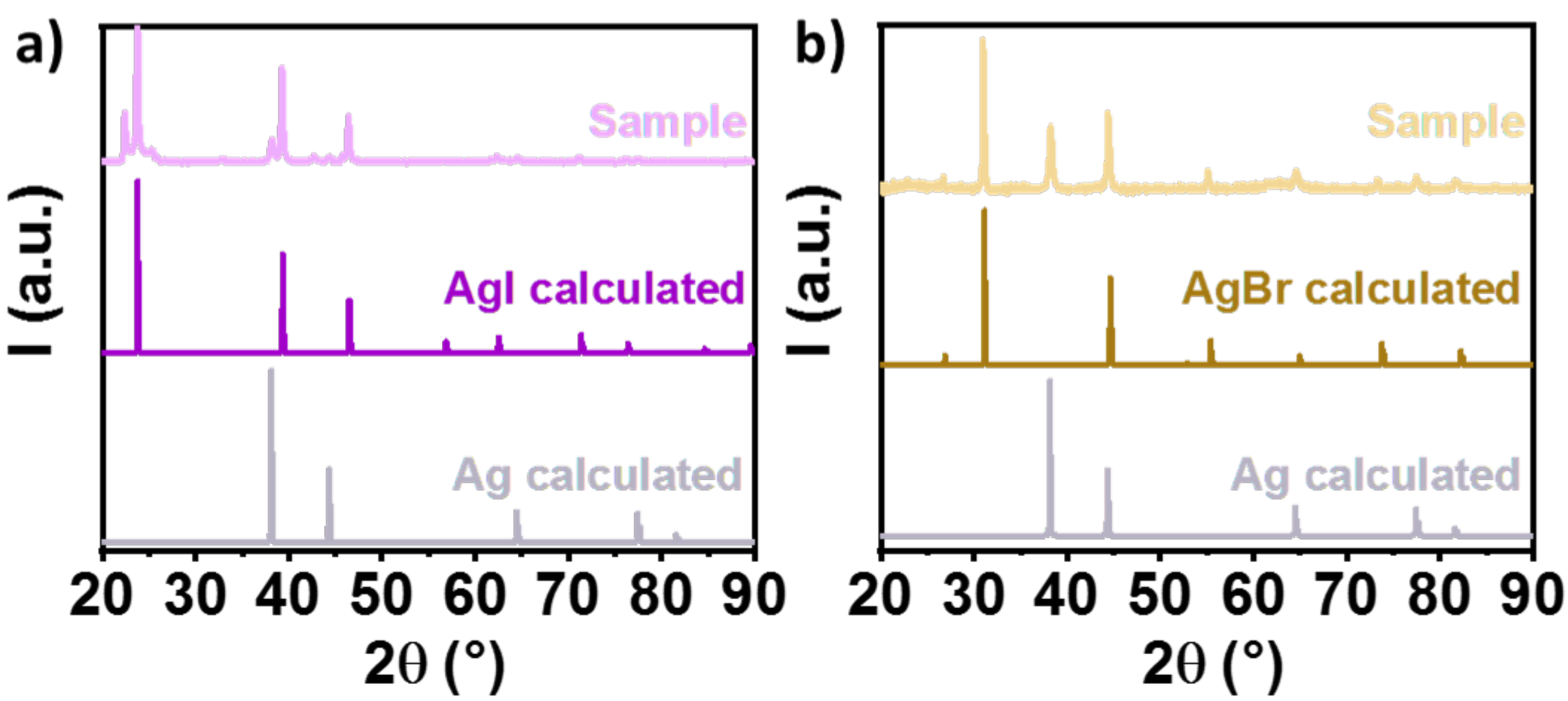

Figure S12: a) Calculated XRD diffractogram of Ag (grey) and AgI (dark purple) and the measured XRD of the Ag@SiO_2 Janus particle reacted with 0.5 mM I_2 (rose) from 2θ of 20 to 90°, b) Calculated XRD diffractogram of Ag (grey) and AgBr (brown) and the measured XRD of the Ag@SiO_2 Janus particle reacted with 0.5 mM Br_2 (yellow) from 2θ of 20 to 90°.

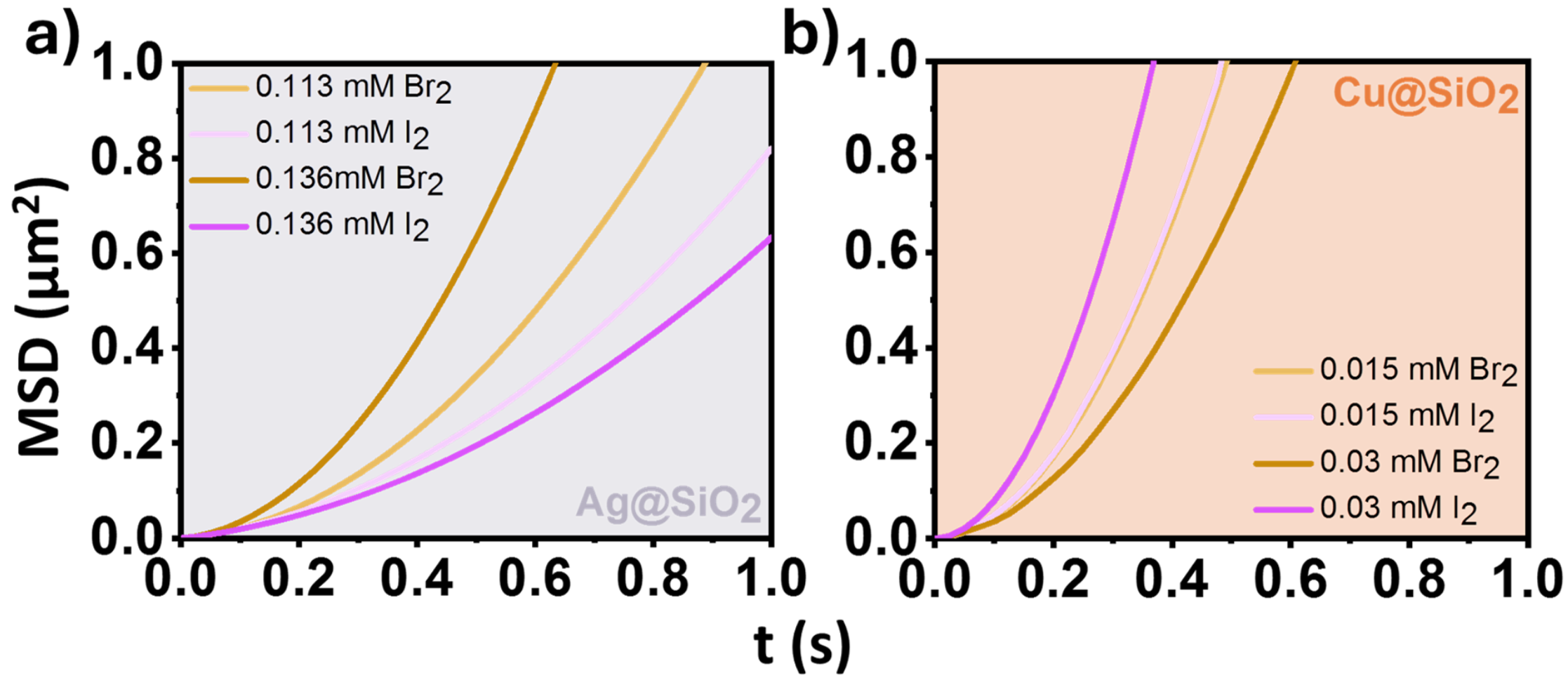

Figure S13: MSD curves for Ag@SiO_2, and Cu@SiO_2 while reacting with halogen solutions such as I_2 and Br_2 at different concentrations.

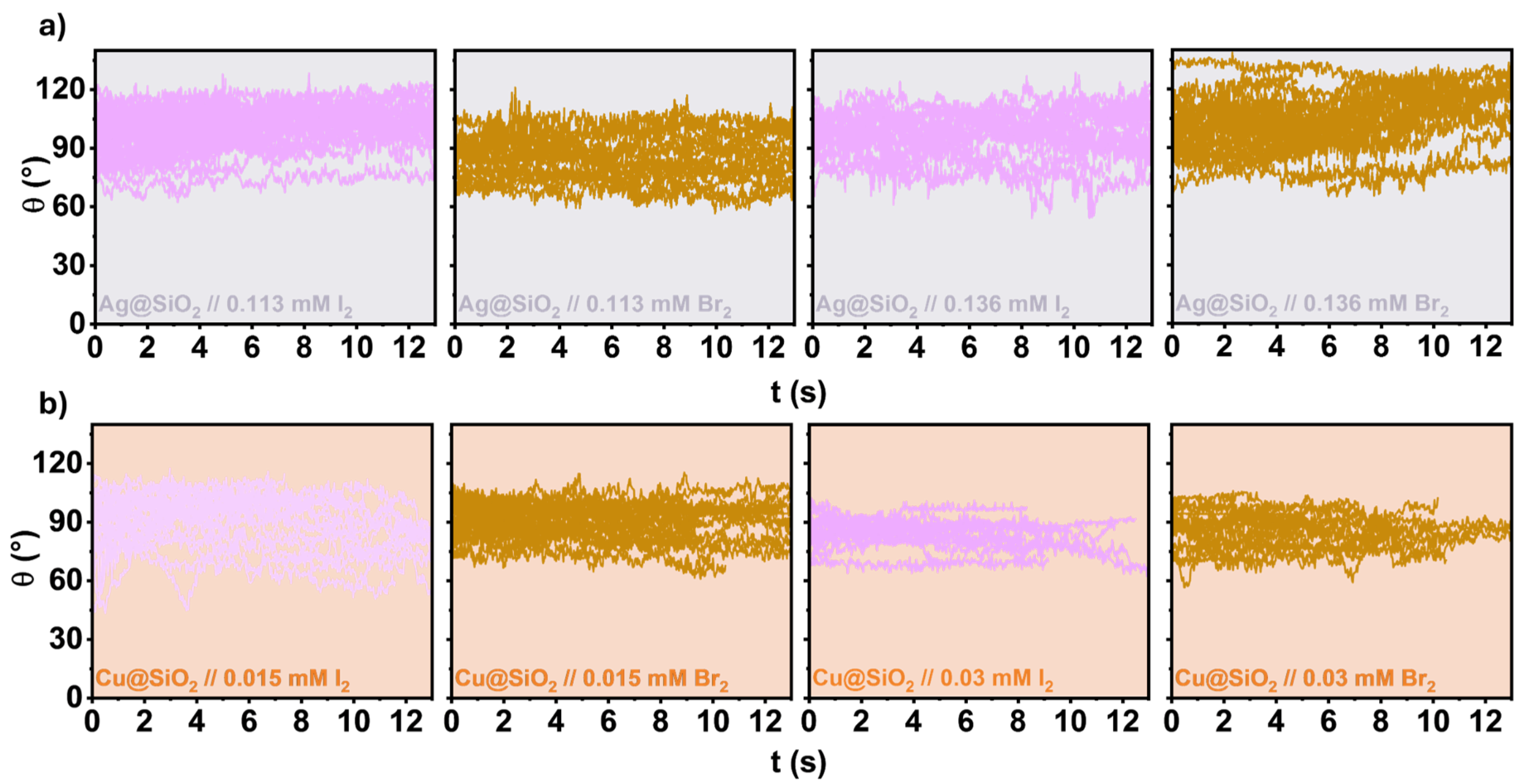

Figure S14: Reorientation angle over time for Ag@SiO_2, and Cu@SiO_2 while reacting with halogen solutions such as I_2 and Br_2 at different concentrations.

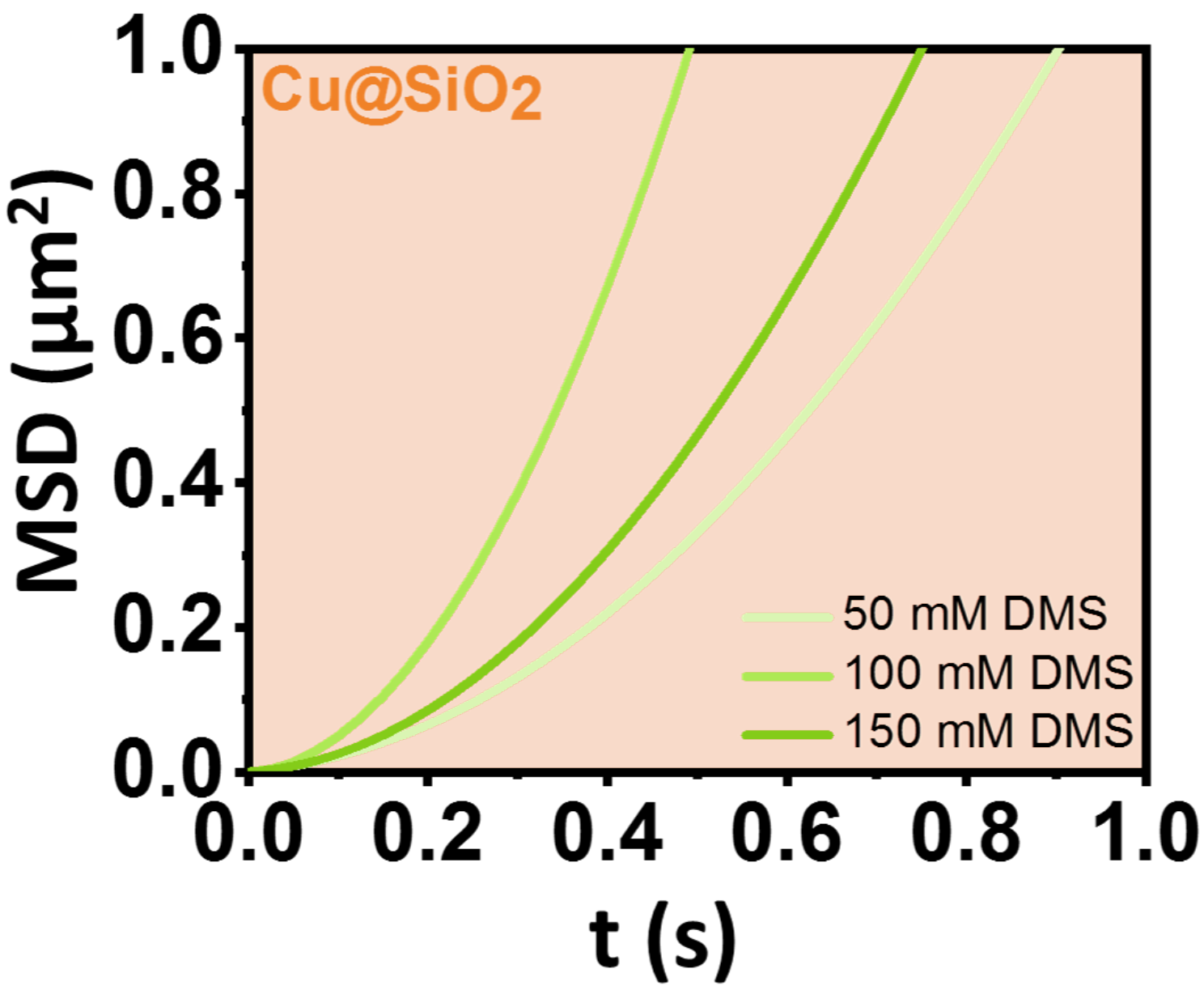

Figure S15: MSD curves for $Cu@SiO_2$ while reacting with DMS at different concentrations.

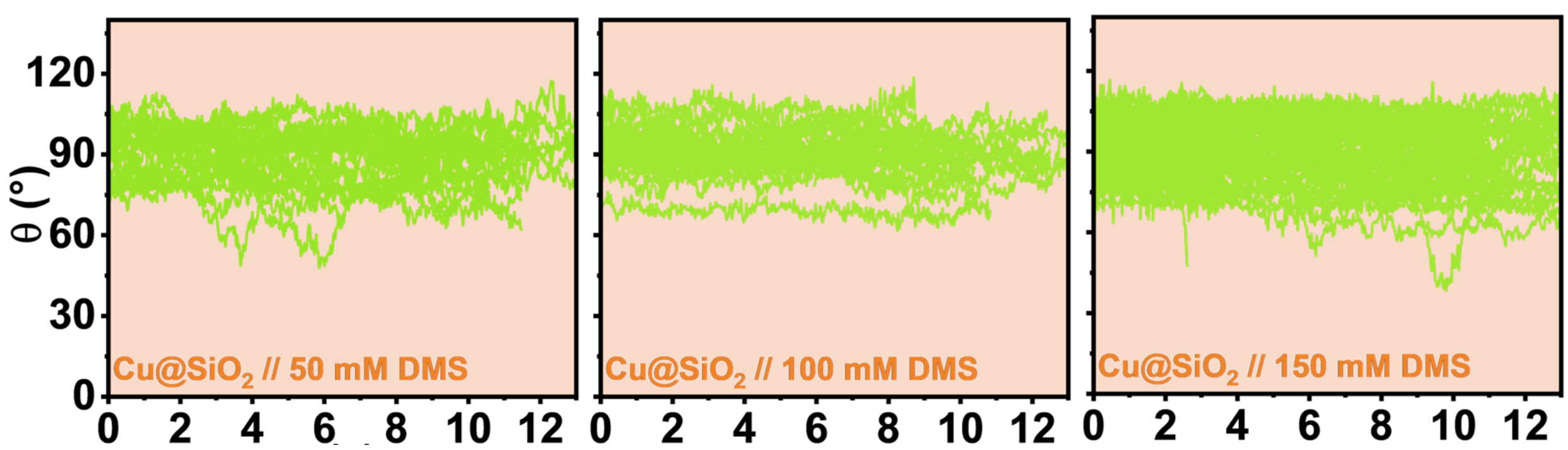

Figure S16: Reorientation angle over time for $Cu@SiO_2$ while reacting with DMS at different concentrations

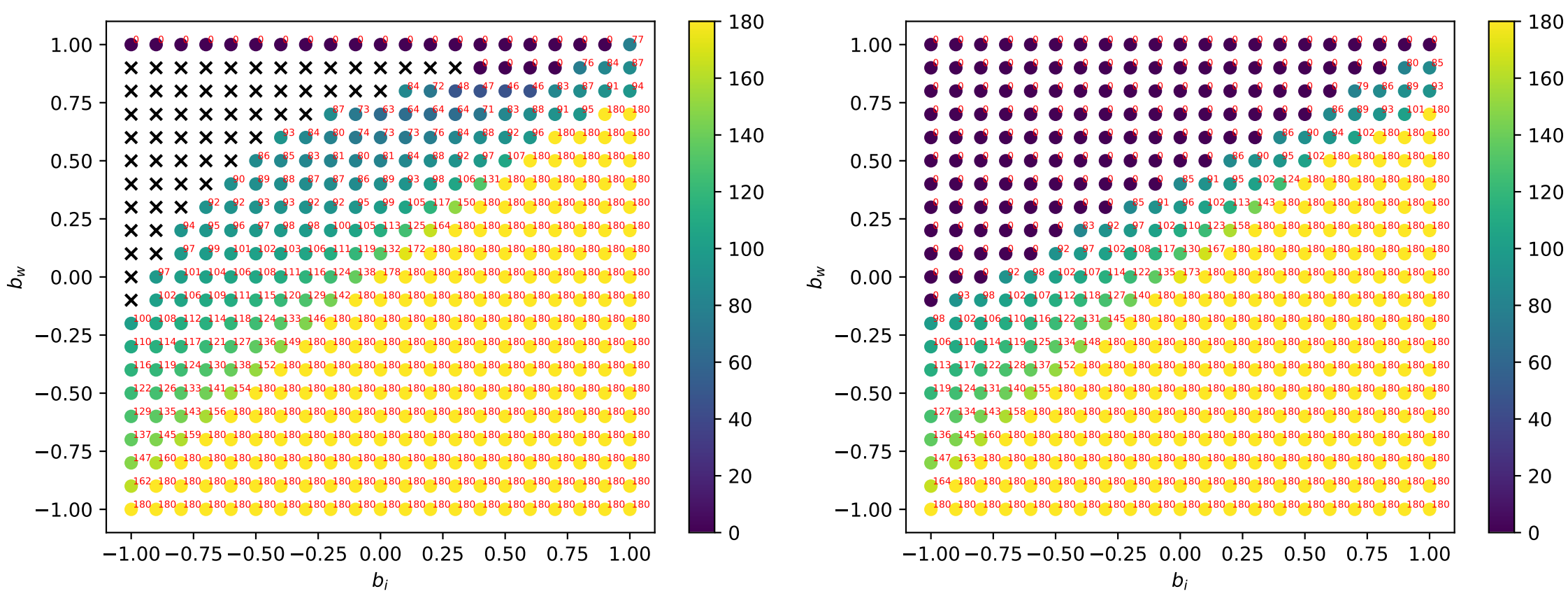

Figure S17: Sliding angles for a particle with $b_c = -1$ for the case of a neutrally buoyant particle (left) and a “heavy” particle (right).

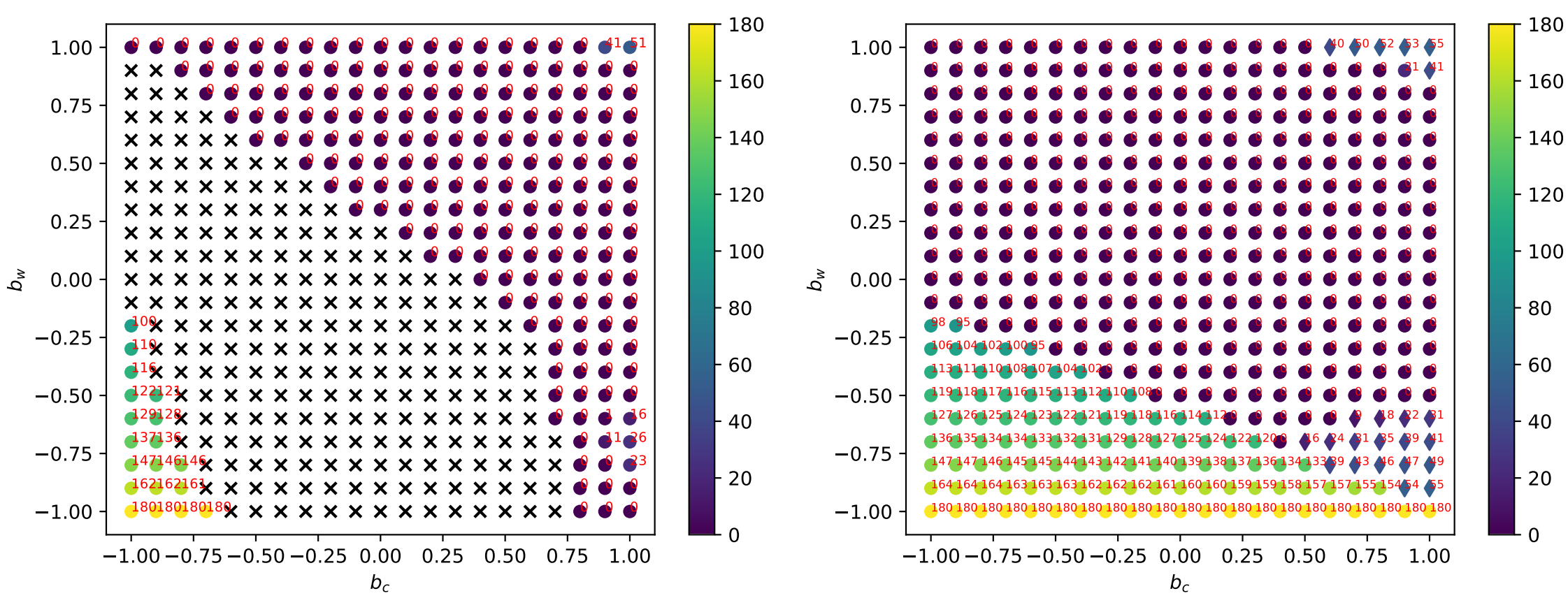

Figure S18: Sliding angles for a particle with $b_i = -1$ for the case of a neutrally buoyant particle (left) and a “heavy” particle (right). Diamond symbols indicate sliding states with motion reversal.

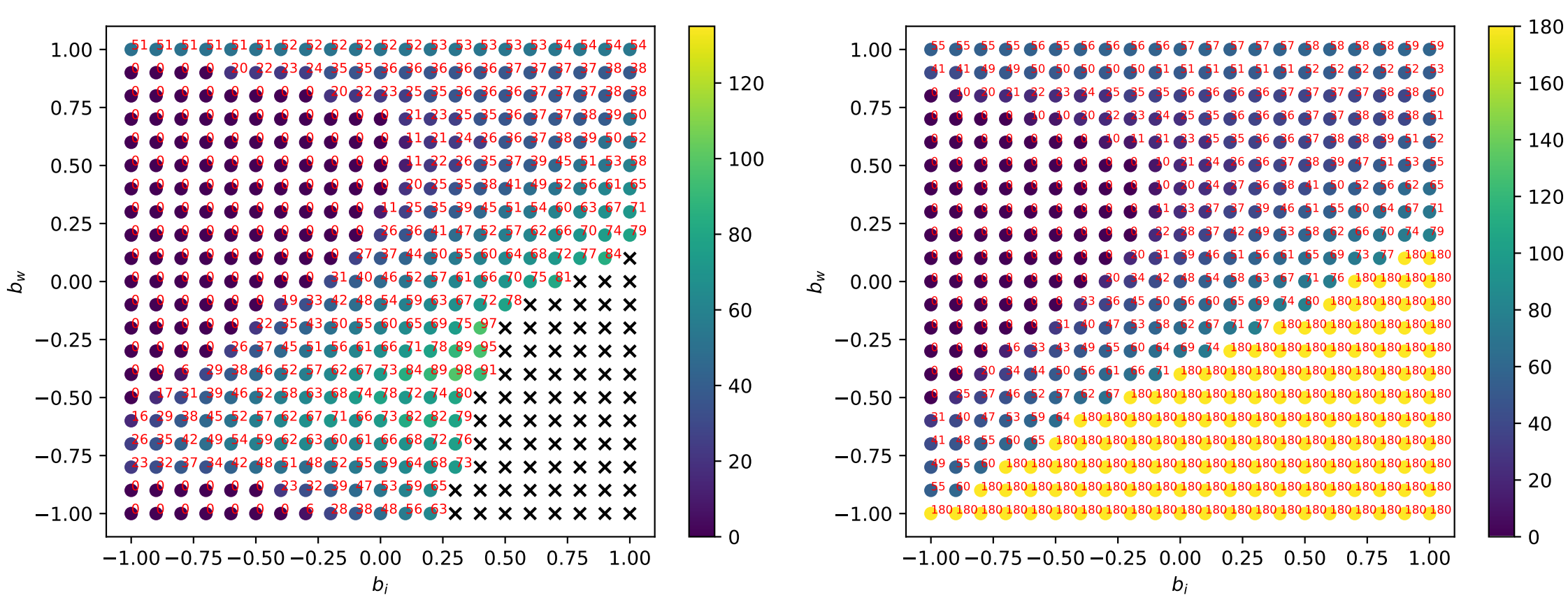

Figure S19: Sliding angles for a particle with $b_c = 1$ for the case of a neutrally buoyant particle (left) and a “heavy” particle (right).

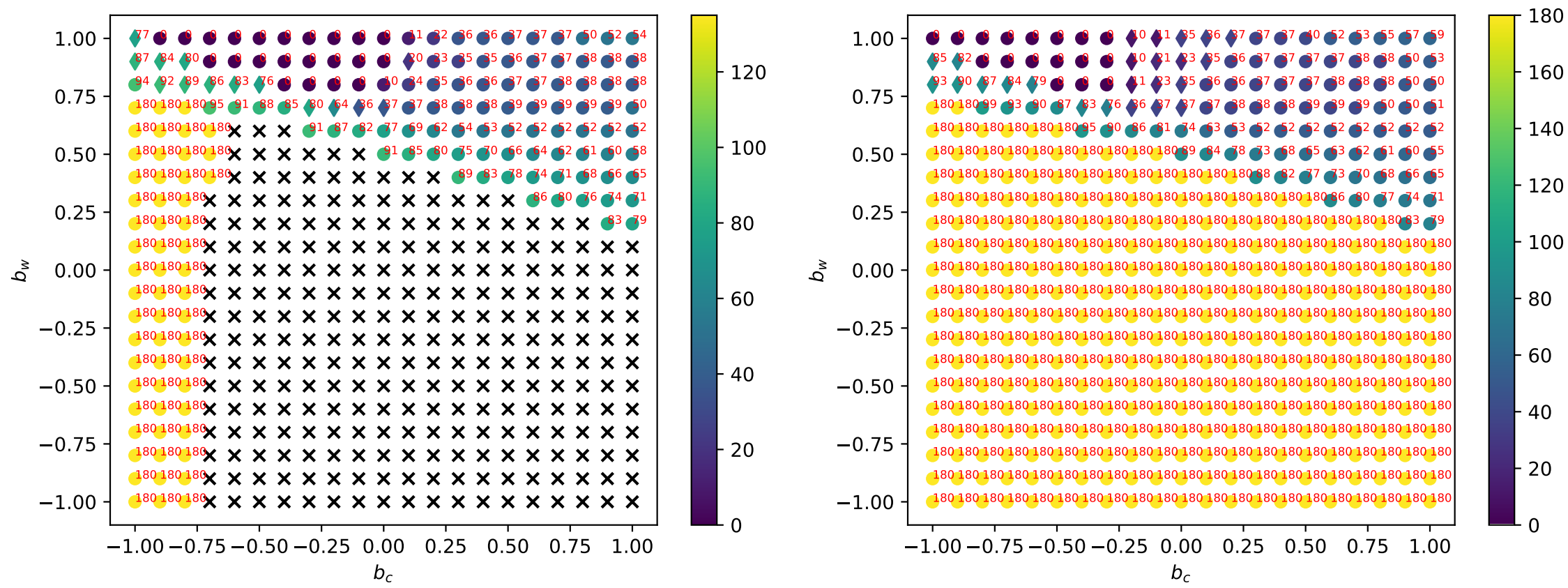

Figure S20: Sliding angles for a particle with $b_i = 1$ for the case of a neutrally buoyant particle (left) and a "heavy" particle (right). Diamond symbols indicate sliding states with motion reversal.

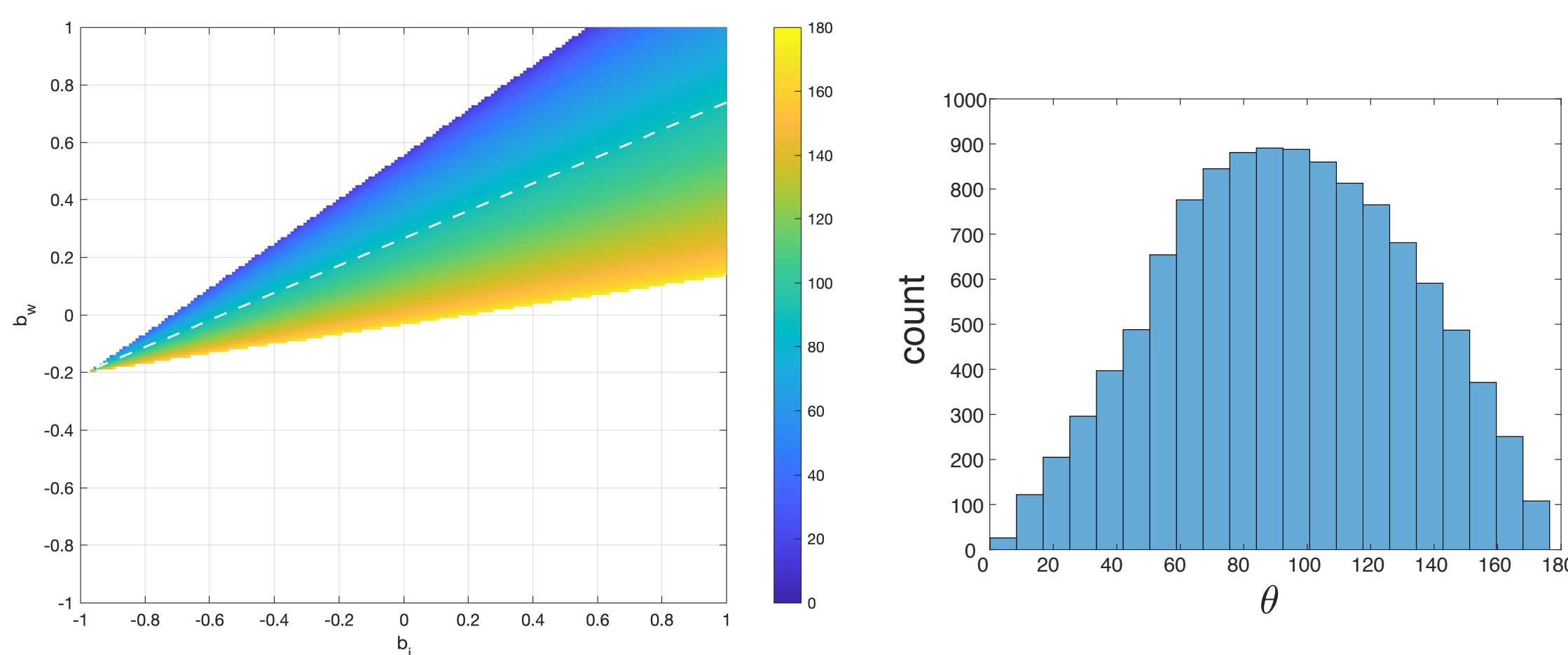

Figure S21: (left) Sliding angles in the analytical model for $b_c = -1$ and with the force quadrupole term omitted ($\gamma_{HI} = 0$) as a function of b_w and b_i. Cap-up and cap-down states are omitted. The white line shows the contour with $\theta = 90°$. (right) Histogram showing the distribution of sliding angles for angles in the range $5° < \theta < 175°$. The distribution is approximately symmetric with respect to $90°$.

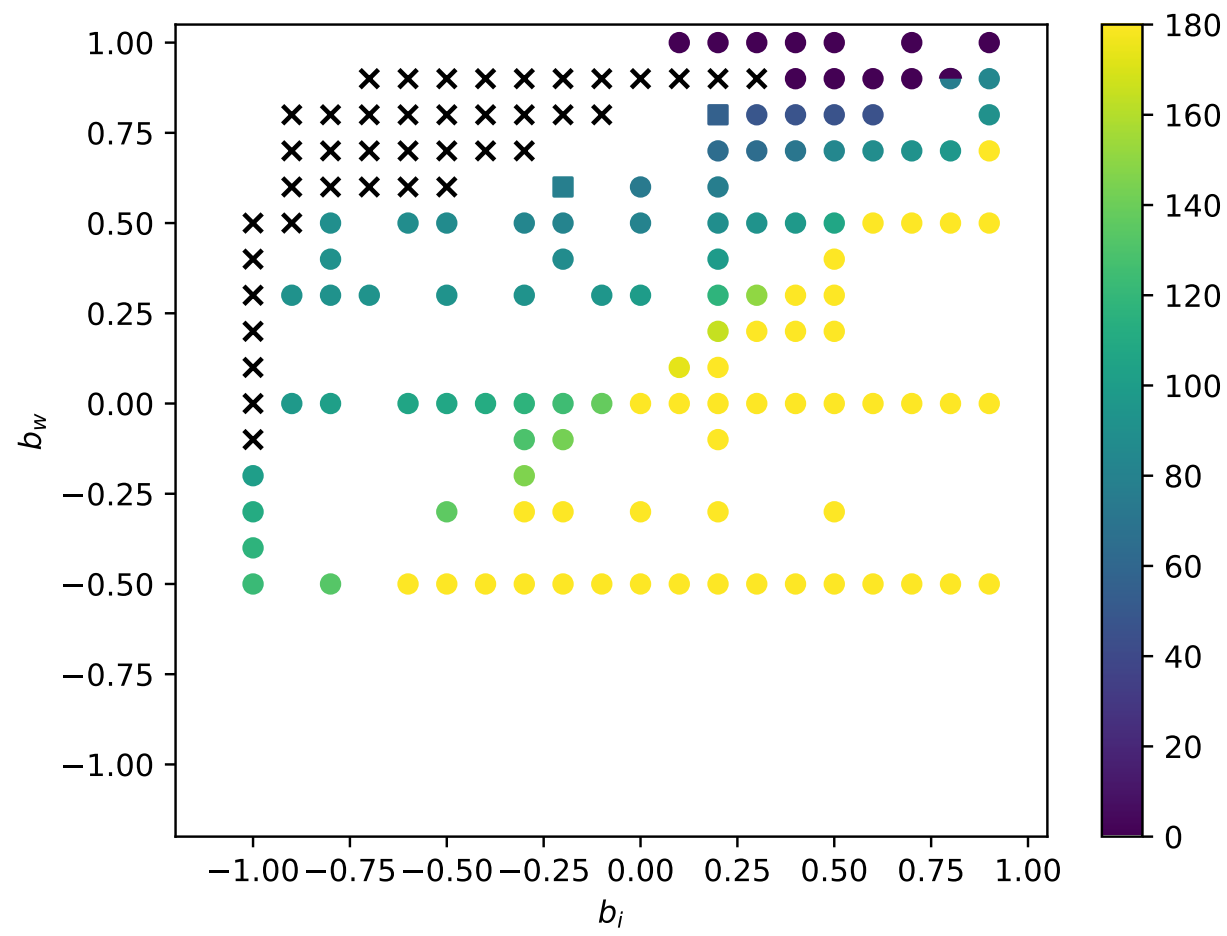

Figure S22: Sliding angles for a particle with $b_c = -1$ for the case of a neutrally buoyant particle. These sliding angles were determined by manual inspection of individual phase portraits. Square symbols indicate limit cycles, with the color showing the cycle-averaged angle. The one symbol with two colors indicates bistablity (two co-existing stable attractors).

Determination of prefactors in analytical model

The analytical model for near-wall sliding has various prefactors associated with wall slip, phoretic rotation, and hydrodynamic interactions. These are determined as follows.

To determine $f_{ws}(h/R)$, where $h/R = 1.1$, we use the maximum absolute value of $\dot{\theta}_{ws}(\theta, h/R = 1.1)$ for $b_w = 1.0$.

To determine $f_{pr}(h/R)$, we calculate $\dot{\theta}_{pr}$ (see Methods) for two parameter combinations. Specifically, we consider the scenarios $(b_i, b_c) = (1, 0)$ and $(b_i, b_c) = (0, 1)$, obtaining $\dot{\theta}_{pr}(\theta, h/R)$ in each case. Numerically, we find $\dot{\theta}_{pr,(1,0)}(\theta, h/R) \approx -\dot{\theta}_{pr,(0,1)}(\theta, h/R)$. This is expected, as a phoretic mobility difference $b_i \neq b_c$ between the two "faces" is required for phoretic rotation. We estimate $f_{pr}(h/R)$ as the maximum absolute value of $\dot{\theta}_{pr,(1,0)}(\theta, h/R = 1.1)$. The full dependence of $\dot{\theta}_{pr}$ on (b_i, b_c) is constructed by linear superposition.

To determine the hydrodynamic singularity strengths α_{HI}, β_{HI}, and γ_{HI}, we first solve the continuum model for an unconfined Janus particle oriented in the $\hat{\mathbf{x}}$ direction with $(b_i, b_c) = (1, 0)$ and $(b_i, b_c) = (0, 1)$. The particle velocity $\mathbf{U}$, surface slip $\mathbf{v}_s(\mathbf{x}_s) = -b\nabla_{||}c$, and

the surface traction $\boldsymbol{\sigma} \cdot \hat{\mathbf{n}}$ obtained by numerical solution of the continuum model are used to calculate the singularity strengths according to Equations 4.14 to 4.16 in Spagnolie and Lauga.[2] Numerically, we find that $\alpha_{\mathrm{HI},(1,0)} \approx -\alpha_{\mathrm{HI},(0,1)}$, $\delta_{\mathrm{HI},(1,0)} \approx \delta_{\mathrm{HI},(0,1)}$, and $\gamma_{\mathrm{HI},(1,0)} \approx \gamma_{\mathrm{HI},(0,1)}$. The full dependence of α_{HI}, β_{HI}, and γ_{HI} on $(\mathrm{b_i}, \mathrm{b_c})$ is constructed by linear superposition.

References

(1) Petr, V. Electrochemical series. *Handbook of Chemistry and Physics* **2012**, 5–80.

(2) Spagnolie, S. E.; Lauga, E. Hydrodynamics of self-propulsion near a boundary: predictions and accuracy of far-field approximations. *Journal of Fluid Mechanics* **2012**, *700*, 105–147.
